# The clinical differentiation of blood culture-positive and -negative sepsis in burn patients: a retrospective cohort study

**DOI:** 10.1093/burnst/tkad031

**Published:** 2023-12-18

**Authors:** Jaechul Yoon, Dohern Kym, Jun Hur, Jongsoo Park, Myongjin Kim, Yong Suk Cho, Wook Chun, Dogeon Yoon

**Affiliations:** Department of Surgery and Critical Care, Burn Center, Hangang Sacred Heart Hospital, Hallym University Medical Center, 12, Beodeunaru-ro 7-gil, Youngdeungpo-gu, Seoul 07247, Korea; Graduate school of Medicine, Kanwon National University, 1, Kangwondaehak-gil, Chuncheon-si, Gangwon-do, Republic of Korea; Burn Institutes, Hangang Sacred Heart Hospital, Hallym University Medical Center, 12, Beodeunaru-ro 7-gil, Youngdeungpo-gu, Seoul 07247, Korea; Department of Surgery and Critical Care, Burn Center, Hangang Sacred Heart Hospital, Hallym University Medical Center, 12, Beodeunaru-ro 7-gil, Youngdeungpo-gu, Seoul 07247, Korea; Burn Institutes, Hangang Sacred Heart Hospital, Hallym University Medical Center, 12, Beodeunaru-ro 7-gil, Youngdeungpo-gu, Seoul 07247, Korea; Department of Surgery and Critical Care, Burn Center, Hangang Sacred Heart Hospital, Hallym University Medical Center, 12, Beodeunaru-ro 7-gil, Youngdeungpo-gu, Seoul 07247, Korea; Burn Institutes, Hangang Sacred Heart Hospital, Hallym University Medical Center, 12, Beodeunaru-ro 7-gil, Youngdeungpo-gu, Seoul 07247, Korea; Department of Surgery and Critical Care, Burn Center, Hangang Sacred Heart Hospital, Hallym University Medical Center, 12, Beodeunaru-ro 7-gil, Youngdeungpo-gu, Seoul 07247, Korea; Burn Institutes, Hangang Sacred Heart Hospital, Hallym University Medical Center, 12, Beodeunaru-ro 7-gil, Youngdeungpo-gu, Seoul 07247, Korea; Department of Surgery and Critical Care, Burn Center, Hangang Sacred Heart Hospital, Hallym University Medical Center, 12, Beodeunaru-ro 7-gil, Youngdeungpo-gu, Seoul 07247, Korea; Burn Institutes, Hangang Sacred Heart Hospital, Hallym University Medical Center, 12, Beodeunaru-ro 7-gil, Youngdeungpo-gu, Seoul 07247, Korea; Department of Surgery and Critical Care, Burn Center, Hangang Sacred Heart Hospital, Hallym University Medical Center, 12, Beodeunaru-ro 7-gil, Youngdeungpo-gu, Seoul 07247, Korea; Burn Institutes, Hangang Sacred Heart Hospital, Hallym University Medical Center, 12, Beodeunaru-ro 7-gil, Youngdeungpo-gu, Seoul 07247, Korea; Department of Surgery and Critical Care, Burn Center, Hangang Sacred Heart Hospital, Hallym University Medical Center, 12, Beodeunaru-ro 7-gil, Youngdeungpo-gu, Seoul 07247, Korea; Burn Institutes, Hangang Sacred Heart Hospital, Hallym University Medical Center, 12, Beodeunaru-ro 7-gil, Youngdeungpo-gu, Seoul 07247, Korea; Burn Institutes, Hangang Sacred Heart Hospital, Hallym University Medical Center, 12, Beodeunaru-ro 7-gil, Youngdeungpo-gu, Seoul 07247, Korea

**Keywords:** Sepsis, Blood culture, Longitudinal, Resistance, Antibiotics, *k*-Means clustering

## Abstract

**Background:**

Sepsis is a potentially life-threatening condition that occurs when the body’s response to infection leads to widespread inflammation and tissue damage. Negative cultures can make it difficult for clinicians to make a diagnosis and may raise questions about the validity of the definition of sepsis. In addition, the clinical distinctions between burn patients with blood culture-positive and -negative sepsis are also poorly understood. Therefore, this study aimed to examine the clinical differences between blood culture-positive and -negative sepsis in burn patients in order to improve the understanding of the pathophysiology and epidemiology of sepsis in this population.

**Methods:**

This study had a retrospective design, and the participants were adults aged ≥18 years. Patients diagnosed with sepsis were divided into two groups based on their blood culture results within 1 week of sepsis diagnosis.

**Results:**

We enrolled 1643 patients admitted to our institution’s burn intensive care unit between January 2010 and December 2021. pH, platelet count, bicarbonate and haematocrit were significant in both the positive and negative groups. However, lymphocyte, red cell distribution width and blood urea nitrogen were significant only in the positive group, whereas lactate dehydrogenase was significant only in the negative group. *Acinetobacter baumannii*, *Pseudomonas aeruginosa*, and *Klebsiella pneumonia* are common gram-negative bacterial species, and *Staphylococcus aureus* and *Staphylococcus epidermidis* are common gram-positive bacterial species seen in burn patients with positive blood cultures. Carbapenem resistance was found to be associated with an unfavourable prognosis in gram-negative bacteria, with the exception of *P. aeruginosa*.

**Conclusions:**

pH, platelet count, bicarbonate and haematocrit were routine biomarkers that demonstrated statistical significance in both groups. Lactate dehydrogenase was significant in the blood-negative group, while red cell distribution width, blood urea nitrogen and lymphocyte count were significant in the positive group. Furthermore, the most common causes of sepsis are gram-negative bacteria, including *A. baumannii*, *K. pneumoniae* and *P. aeruginosa*. Additionally, resistance to carbapenems is associated with unfavourable outcomes.

Highlights

- This study examined clinical differences between burn patients with blood culture-positive and -negative sepsis to improve understanding of the pathophysiology and epidemiology of sepsis in this population.
- The study found that pH, platelet count, bicarbonate level and hematocrit level were significant biomarkers in both blood culture-positive and -negative sepsis patients, while LD was significant only in the negative group, and lymphocyte and blood urea nitrogen were significant only in the positive group.
- *Acinetobacter baumannii*, *Pseudomonas aeruginosa* and *Klebsiella pneumoniae* were identified as common gram-negative bacterial species, and *Staphylococcus aureus* and *Staphylococcus epidermidis* as common gram-positive bacterial species in burn patients with positive blood cultures. Carbapenem resistance was associated with unfavourable prognosis in gram-negative bacteria, except in *P. aeruginosa*.
- The study utilized a deep learning mechanism and the largest available dataset to identify meaningful biomarkers in sepsis without human selection bias, but the single-centre study may be subject to geographical bias, limiting the generalizability of the findings.

## Background

Sepsis is a potentially life-threatening condition that occurs when the body’s response to infection leads to widespread inflammation and tissue damage. In burn patients, sepsis can develop from the burn injury itself or from an infection that occurs at the burn site. The mortality rate from sepsis remains high at 50–60% [1]. The diagnostic criteria for sepsis are defined as the presence of suspected or documented infection, along with the presence of at least two of the four clinical criteria for systemic inflammatory response syndrome (SIRS), as defined by the American College of Chest Physicians (ACCP) and the Society of Critical Care Medicine in 1991 [2]. However, clinicians usually experience challenges in diagnosing sepsis using traditional SIRS criteria because of burn patients’ vulnerability to infection due to skin damage. In 2007, burn sepsis, a term used specifically for burn patients, was defined by the Consensus Conference to Define Sepsis and Infection in Burns by creating burn SIRS criteria specialized for burn patients [3]. In 2015, the Sequential Organ Failure Assessment (SOFA) score was defined by the Society of Critical Care Medicine and the European Society of Intensive Care Medicine as an increase of at least 2 points in the presence of suspected or obvious infection [4]. Research demonstrating the effectiveness of Sepsis-3 in burn patients has been published [5,6]. However, patients with burns are always at risk of developing sepsis, which can lead to high heterogeneity. While early detection and treatment of sepsis are crucial for improving outcomes in burn patients, there are also clinical differences between culture-positive and culture-negative sepsis that healthcare providers should be aware of since negative cultures can make it difficult for clinicians to make a diagnosis and may raise questions about the validity of the definition of sepsis [7]. In addition, the presence or absence of positive and negative cultures may be fundamentally different in relation to the pathophysiology, epidemiology and treatment response observed in burn patients [8]. Besides, the clinical distinctions between patients with blood culture-positive and -negative sepsis are not well understood because the mortality and clinical outcomes of patients with blood culture-positive and -negative sepsis have been inconsistently reported, leading to controversy. Therefore, this study aimed to examine the clinical differences between burn patients with blood culture-positive and -negative sepsis in order to improve the understanding of the pathophysiology and epidemiology of sepsis in this population. We hypothesized that by gaining a better understanding of these differences we might improve the diagnosis and treatment of sepsis in patients with burns.

## Methods

### Study site and patients

**Figure 1:**
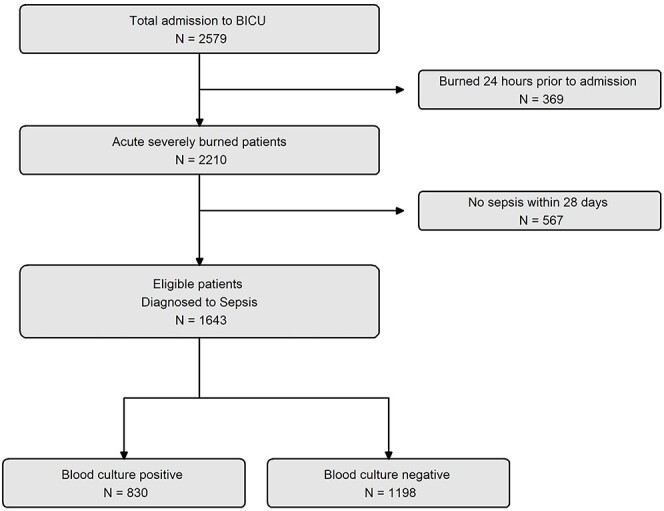
Flowchart of enroled patients. *BICU* burn intensive care unit

We used a retrospective design, and the participants were adult patients with burns aged ≥18 years who were admitted to the burn intensive care unit (BICU) of our institution between November 2010 and December 2021. Patients diagnosed with sepsis were divided into two groups based on their blood culture results within 1 week of their diagnosis, as blood culture results typically take at least 3 days to be obtained. All patients in both groups underwent routine laboratory tests, including complete blood counts, arterial blood gas analyses, electrolyte levels and blood chemistry, performed at least every 3–4 days during their intensive care unit (ICU) stay. A total of 2579 patients were admitted to the BICU between January 2010 and December 2021. We excluded patients who had been hospitalized for >2 days following their burns. Among the remaining 2210 patients, 567 did not develop sepsis, and the remaining 1643 patients were divided into two groups based on the presence or absence of blood culture results within 1 week of sepsis diagnosis, i.e. blood culture-positive and -negative groups. Out of 1643 patients, 345 experienced both positive and negative sepsis test results during the study period. The study enrolment process is depicted in Figure 1.

### Data collection and missing values

The clinical database warehouse (CDW) of our institution provided us with a comprehensive set of longitudinal clinical data collected through a prospective approach. All relevant variables were gathered starting at the point of admission and continuing until the time of death in the non-survival group and until discharge from the BICU in the survival group. The worst biomarker values were obtained when measurements were taken multiple times per day. Demographic characteristics, such as patient age, sex and extent of burns, were determined using modified Lund and Browder charts. Additional factors considered included the type of burn, length of stay in the BICU, presence of inhalation injury and routine laboratory test results from the CDW. After admission, the severity of the injury was assessed using the Abbreviated Burn Severity Index (ABSI), revised Baux (rBaux) index and our newly developed score parameter (Hangang) [9]. Acute Physiology and Chronic Health Evaluation Score (APACHE) IV and SOFA scores were calculated daily using routine laboratory results. The missing values for these longitudinal variables were imputed using the copyMean method, a commonly used method to predict missing data in longitudinal studies [10]. This method involves interpolating the values surrounding the missing data using a straight line and imputing the missing values using the last observation carried forward or following observation carried backward methods.

### Outcome, sepsis diagnosis, infection identification and antibiotic administration

The primary outcome was the in-hospital mortality rate within 60 days. Sepsis was diagnosed based on the SOFA score, with a score ≥2 according to the Sepsis-3 criteria indicating the presence of an acute change that is considered abnormal [4]. To identify infections, we defined a suspected infection as a situation in which cultures were obtained from blood, wounds, sputum or urine before the results were obtained, and antibiotics were administered. On the other hand, a documented infection was defined as a confirmed infection through positive culture results or pathological tissue sources. According to our protocol for blood cultures, blood samples should be collected from two different sites during fever, and antibiotic administration should occur after obtaining the blood samples, except in certain critical situations where physician discretion is required. The prompt initiation of empirical broad-spectrum antibiotics, aimed at the most likely pathogens considering the patient’s clinical presentation, underlying conditions and local antimicrobial resistance patterns, was carried out within an hour of acquiring culture samples.

### Statistical analysis

The basic demographic characteristics are described as follows: continuous numerical variables with a normal distribution are presented as mean ± standard deviation (SD), while those with a non-normal distribution are presented as medians and interquartile range (IQR). Depending on the normality of the distribution, an independent t-test or Wilcoxon signed-rank test was used to determine the differences between the two groups. Categorical variables were expressed as percentages and compared between groups using the chi-square test or Fisher’s exact test, as appropriate. Longitudinal biomarkers were grouped into three clusters using the *k*-means clustering algorithm, which is an effective method for clustering longitudinal data based on their shape. Each cluster was assigned a letter from A to C, according to its mortality rate, and this was performed using the kmlShape package in the R-project program [11]. Univariate and multivariate logistic regression analyses were also performed. Furthermore, we removed biomarkers that were highly correlated with each other (multicollinearity) and used a stepwise selection method to select the final set of biomarkers for our analysis. All tests were two-tailed, and *p*-values < 0.05 were considered to be statistically significant. All analyses were performed using the Statistical R-project program version 4.2.2.

**Table 1:** Demographics for all sepsis patients

**Group**	**Variables**	**Overall, n = 1643**	**Survivors, n = 1214 (73.9%)**	**Non-survivors, n = 429 (26.1%)**	***P* value**
Demographics	Patient age (years)				<0.001
	Median [IQR]	52 [42, 63]	51 [41, 61]	55 [45, 70]	
	Sex, n (%)				0.819
	Male	1297 (78.9%)	960 (79.1%)	337 (78.6%)	
	Female	346 (21.1%)	254 (20.9%)	92 (21.4%)	
	Type, n (%)				<0.001
	FB	1229 (74.8%)	870 (71.7%)	359 (83.7%)	
	SB	151 (9.2%)	115 (9.5%)	36 (8.4%)	
	EB	154 (9.4%)	143 (11.8%)	11 (2.6%)	
	ChB	21 (1.3%)	17 (1.4%)	4 (0.9%)	
	CoB	88 (5.4%)	69 (5.7%)	19 (4.4%)	
	TBSA				<0.001
	Median [IQR]	33 [20, 51]	28 [17, 41]	60 [37, 80]	
	Inhalation	718 (43.7%)	483 (39.8%)	235 (54.8%)	<0.001
	LOICU				<0.001
	Median [IQR]	22 [11, 36]	24 [12, 40]	16 [10, 25]	
Severity scores	ABSI				<0.001
	Median [IQR]	8 [7, 10]	8 [7, 9]	11 [9, 13]	
	rBaux				<0.001
	Median [IQR]	95 [78, 114]	88 [74, 102]	121 [105, 141]	
	Hangang				<0.001
	Median [IQR]	130 [118, 144]	125 [115, 135]	152 [140, 164]	
	APACHE IV				<0.001
	Median [IQR]	44 [33, 60]	39 [30, 53]	59 [45, 77]	
	SOFA				<0.001
	Median [IQR]	4 [3, 6]	4 [3, 5]	6 [5, 8]	
Comorbidities	Hypertension, n (%)	308 (18.7%)	205 (16.9%)	103 (24.0%)	0.001
	Diabetes mellitus, n (%)	157 (9.6%)	104 (8.6%)	53 (12.4%)	0.022
	Tuberculosis, n (%)	27 (1.6%)	19 (1.6%)	8 (1.9%)	0.675
	Hepatobiliary, n (%)	36 (2.2%)	29 (2.4%)	7 (1.6%)	0.357
	Cardiovascular, n (%)	47 (2.9%)	35 (2.9%)	12 (2.8%)	0.927
	CVA, n (%)	30 (1.8%)	22 (1.8%)	8 (1.9%)	0.944
	Cancer, n (%)	35 (2.1%)	23 (1.9%)	12 (2.8%)	0.266
	Hyperlipidemia, n (%)	46 (2.8%)	36 (3.0%)	10 (2.3%)	0.494
	Other, n (%)	445 (27.1%)	336 (27.7%)	109 (25.4%)	0.363

*IQR* interquartile range, *FB* flame burn, *SB* scale burn, *EB* electrical burn, *ChB* chemical burn, *CoB* contact burn, *LOICU* length of ICU stay, *ABSI* Abbreviated Burn Severity Index, *rBaux* revised Baux index, *APACHE* Acute Physiology and Chronic Health Evaluation score, *SOFA* Sequential Organ Failure Assessment score, *CVA* cerebrovascular accident.

## Results

### Baseline characteristics of study populations

**Figure 2:**
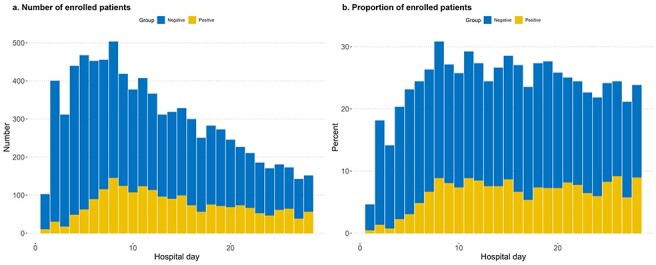
Number (**a**) and proportion (**b**) of enrolled patients with sepsis

Among the eligible patients, 429 (26.1%) died. The overall median age was 52 (42–63) years, with an age range of 18 to 99 years. The study population had a male predominance, with 78.9% of the patients being male. In addition, the median total body surface area (TBSA) affected was 33%, and inhalation injuries were present in 718 (43.7%) patients. Moreover, the median length of stay in the ICU was 22 (11–36) days, and the APACHE IV, SOFA, ABSI, rBaux and our newly developed parameter scores were 44, 4, 8, 95 and 130, respectively. Furthermore, the most common comorbidities recorded were hypertension (18.7%), diabetes mellitus (9.6%), cardiovascular disease (2.9%) and hyperlipidemia (2.8%) (Table 1). However, in the blood culture-positive group, 310 (37.3%) patients died, and their median age (IQR) was 52 (42–63) years, with a male predominance of 79.0%. In addition, their median TBSA affected was 42%, and inhalation injuries were present in 401 (48.3%) patients. Further, the median length of their stay in the ICU was 27 (17–43) days, and their median APACHE IV, SOFA, ABSI, rBaux, and our newly developed parameter scores were 46, 4, 9, 106 and 132, respectively. Notably, the most common comorbidities recorded in this group were hypertension (18.8%), diabetes mellitus (9.6%), hyperlipidemia (2.4%) and cardiovascular disease (2.2%) (Table 2). In contrast, in the blood culture-negative group, 237 (19.8%) patients died, and their median age (IQR) was 52 (42–64) years, with a male predominance of 77.7%. In addition, their median TBSA was 29% and inhalation injuries were present in 486 (40.6%) patients (Table 2). We found that the number and proportion of sepsis cases according to the number of days in the hospital were higher in the blood culture-negative group than in the blood culture-positive group (Figure 2).

**Table 2:** Demographics for blood culture positive/negative groups

		**Blood positive**				**Blood negative**			
**Group**	**Variables**	**Overall, n = 830**	**Survivors, n = 520 (62.7%)**	**Non-survivors, n = 310 (37.3%)**	***P* value**	**Overall, n = 1198**	**Survivors, n = 961 (80.2%)**	**Non-survivors, n = 237 (19.8%)**	***P* value**
Demographics	Patient age				0.004				<0.001
	Median [IQR]	52 [42, 63]	52 [42, 61]	54 [43, 66]		52 [42, 64]	51 [41, 62]	59 [48, 74]	
	Sex, n (%)				0.723				0.282
	Male	656 (79.0%)	413 (79.4%)	243 (78.4%)		931 (77.7%)	753 (78.4%)	178 (75.1%)	
	Female	174 (21.0%)	107 (20.6%)	67 (21.6%)		267 (22.3%)	208 (21.6%)	59 (24.9%)	
	Type				0.002				<0.001
	FB	650 (78.3%)	391 (75.2%)	259 (83.5%)		870 (72.6%)	677 (70.4%)	193 (81.4%)	
	SB	78 (9.4%)	49 (9.4%)	29 (9.4%)		111 (9.3%)	90 (9.4%)	21 (8.9%)	
	EB	59 (7.1%)	50 (9.6%)	9 (2.9%)		125 (10.4%)	121 (12.6%)	4 (1.7%)	
	ChB	11 (1.3%)	9 (1.7%)	2 (0.6%)		15 (1.3%)	12 (1.2%)	3 (1.3%)	
	CoB	32 (3.9%)	21 (4.0%)	11 (3.5%)		77 (6.4%)	61 (6.3%)	16 (6.8%)	
	TBSA				<0.001				<0.001
	Median [IQR]	42 [30, 61]	37 [25, 50]	60 [40, 80]		29 [16, 44]	25 [15, 39]	52 [30, 74]	
	Inhalation, n (%)	401 (48.3%)	240 (46.2%)	161 (51.9%)	0.107	486 (40.6%)	367 (38.2%)	119 (50.2%)	<0.001
	LOICU				<0.001				<0.001
	Median [IQR]	27 [17, 43]	36 [23, 52]	18 [12, 25]		21 [10, 36]	22 [11, 38]	17 [10, 26]	
Severity scores	ABSI				<0.001				<0.001
	Median [IQR]	9 [8, 11]	9 [8, 10]	11 [9, 13]		8 [7, 9]	8 [6, 9]	11 [9, 13]	
	rBaux				<0.001				<0.001
	Median [IQR]	106 [90, 122]	98 [84, 111]	122 [107, 139]		90 [75, 107]	86 [72, 99]	117 [99, 140]	
	Hangang				<0.001				<0.001
	Median [IQR]	132 [120, 146]	125 [117, 135]	148 [138, 160]		126 [117, 140]	123 [115, 133]	150 [139, 162]	
	APACHE IV				<0.001				<0.001
	Median [IQR]	46 [33, 63]	41 [30, 53]	59 [43, 76]		43 [31, 59]	38 [28, 52]	62 [49, 78]	
	SOFA				<0.001				<0.001
	Median [IQR]	4 [3, 6]	4 [3, 5]	6 [5, 9]		4 [3, 6]	4 [3, 5]	7 [5, 9]	
Comorbidities	Hypertension, n (%)	156 (18.8%)	91 (17.5%)	65 (21.0%)	0.216	227 (18.9%)	167 (17.4%)	60 (25.3%)	0.005
	Diabetes mellitus, n (%)	80 (9.6%)	44 (8.5%)	36 (11.6%)	0.137	116 (9.7%)	86 (8.9%)	30 (12.7%)	0.084
	Tuberculosis, n (%)	11 (1.3%)	6 (1.2%)	5 (1.6%)	0.550	22 (1.8%)	16 (1.7%)	6 (2.5%)	0.415
	Hepatobiliary, n (%)	16 (1.9%)	11 (2.1%)	5 (1.6%)	0.611	28 (2.3%)	23 (2.4%)	5 (2.1%)	0.796
	Cardiovascular, n (%)	18 (2.2%)	9 (1.7%)	9 (2.9%)	0.262	37 (3.1%)	31 (3.2%)	6 (2.5%)	0.580
	CVA, n (%)	14 (1.7%)	9 (1.7%)	5 (1.6%)	0.899	25 (2.1%)	18 (1.9%)	7 (3.0%)	0.310
	Cancer, n (%)	20 (2.4%)	10 (1.9%)	10 (3.2%)	0.236	26 (2.2%)	19 (2.0%)	7 (3.0%)	0.355
	Hyperlipidemia, n (%)	20 (2.4%)	14 (2.7%)	6 (1.9%)	0.492	37 (3.1%)	31 (3.2%)	6 (2.5%)	0.580
	Other, n (%)	214 (25.8%)	140 (26.9%)	74 (23.9%)	0.331	339 (28.3%)	271 (28.2%)	68 (28.7%)	0.880

*IQR* interquartile range, *FB* flame burn, *SB* scale burn, *EB* electrical burn, *ChB* chemical burn, *CoB* contact burn, *LOICU* length of ICU stay, *ABSI* Abbreviated Burn Severity Index, *rBaux* revised Baux index, *APACHE* Acute Physiology and Chronic Health Evaluation score, *SOFA* Sequential Organ Failure Assessment score, *CVA* cerebrovascular accident

### Predictors for both groups

We evaluated 24 biomarkers that were checked at least every 4 days. In the blood culture-positive group, seven biomarkers (i.e. pH, platelet count, bicarbonate, haematocrit, red cell distribution width (RDW), lymphocyte count and blood urea nitrogen (BUN) level were statistically significant. However, in the blood culture-negative group, five biomarkers (i.e. pH, platelet count, bicarbonate, haematocrit and lactate dehydrogenase [LD]) were statistically significant. It is worth noting that four biomarkers were statistically significant in both groups (Table 3). The kmlShape package was utilized to generate longitudinal plots for each cluster, as demonstrated in Figures S1–S8, see online supplementary maerial. The characteristics and temporal variations in value for eight significant biomarkers are also summarized in Tables S1–S8, see online supplementary material.

**Table 3:** Multiple logistic regression for mortality in both groups

	**Blood positive**		**Blood negative**	
**Characteristic**	**OR (95% CI)**	***P* value**	**OR (95% CI)**	***P* value**
Age	1.034 (1.013, 1.055)	**0.001**	1.075 (1.055, 1.097)	**<0.001**
TBSA	1.039 (1.024, 1.055)	**<0.001**	1.061 (1.046, 1.077)	**<0.001**
Inhalation		**0.007**		**0.032**
No	2.200 (1.237, 3.989)		1.851 (1.053, 3.301)	
Ventilator period	1.265 (1.105, 1.459)	**<0.001**	1.521 (1.372, 1.697)	**<0.001**
CRRT period			1.354 (1.128, 1.633)	**<0.001**
pH		**<0.001**		**<0.001**
Cluster A	Reference		Reference	
Cluster B	2.045 (1.024, 4.129)		1.699 (0.854, 3.484)	
Cluster C	29.43 (6.954, 163.0)		4.521 (1.965, 10.69)	
Platelet		**<0.001**		**0.006**
Cluster A	Reference		Reference	
Cluster B	2.411 (0.814, 8.611)		0.740 (0.267, 2.223)	
Cluster C	7.716 (2.598, 27.58)		2.039 (0.765, 6.008)	
Bicarbonate		**0.007**		**0.012**
Cluster A	Reference		Reference	
Cluster B	2.317 (1.147, 4.794)		2.555 (1.295, 5.238)	
Cluster C	3.800 (1.644, 9.041)		2.921 (1.337, 6.534)	
Hct		**0.012**		**0.001**
Cluster A	Reference		Reference	
Cluster B	1.338 (0.581, 3.158)		0.622 (0.264, 1.444)	
Cluster C	2.676 (1.184, 6.300)		2.171 (1.143, 4.215)	
RDW		**0.003**		0.082
Cluster A	Reference		Reference	
Cluster B	2.278 (1.163, 4.607)		1.206 (0.594, 2.514)	
Cluster C	4.407 (1.839, 10.90)		2.395 (1.004, 5.822)	
Lymphocyte		**0.002**		
Cluster A	Reference		Reference	
Cluster B	0.716 (0.310, 1.688)			
Cluster C	1.894 (0.829, 4.424)			
BUN		**0.048**		
Cluster A	Reference		Reference	
Cluster B	0.529 (0.271, 1.020)			
Cluster C	1.048 (0.486, 2.244)			
LD		0.054		**0.004**
Cluster A	Reference		Reference	
Cluster B	1.237 (0.714, 2.151)		1.457 (0.838, 2.542)	
Cluster C	3.880 (1.282, 12.44)		3.733 (1.713, 8.260)	

The *p* value was denoted in bold font to indicate statistical significance *<*0.05

*OR* odds ratio, *CI* confidence interval, *CRRT* continuous renal replacement therapy, *Hct* hematocrit, *RDW* red cell distribution width, *BUN* blood urea nitrogen, *LD* lactate dehydrogenase

**Figure 3:**
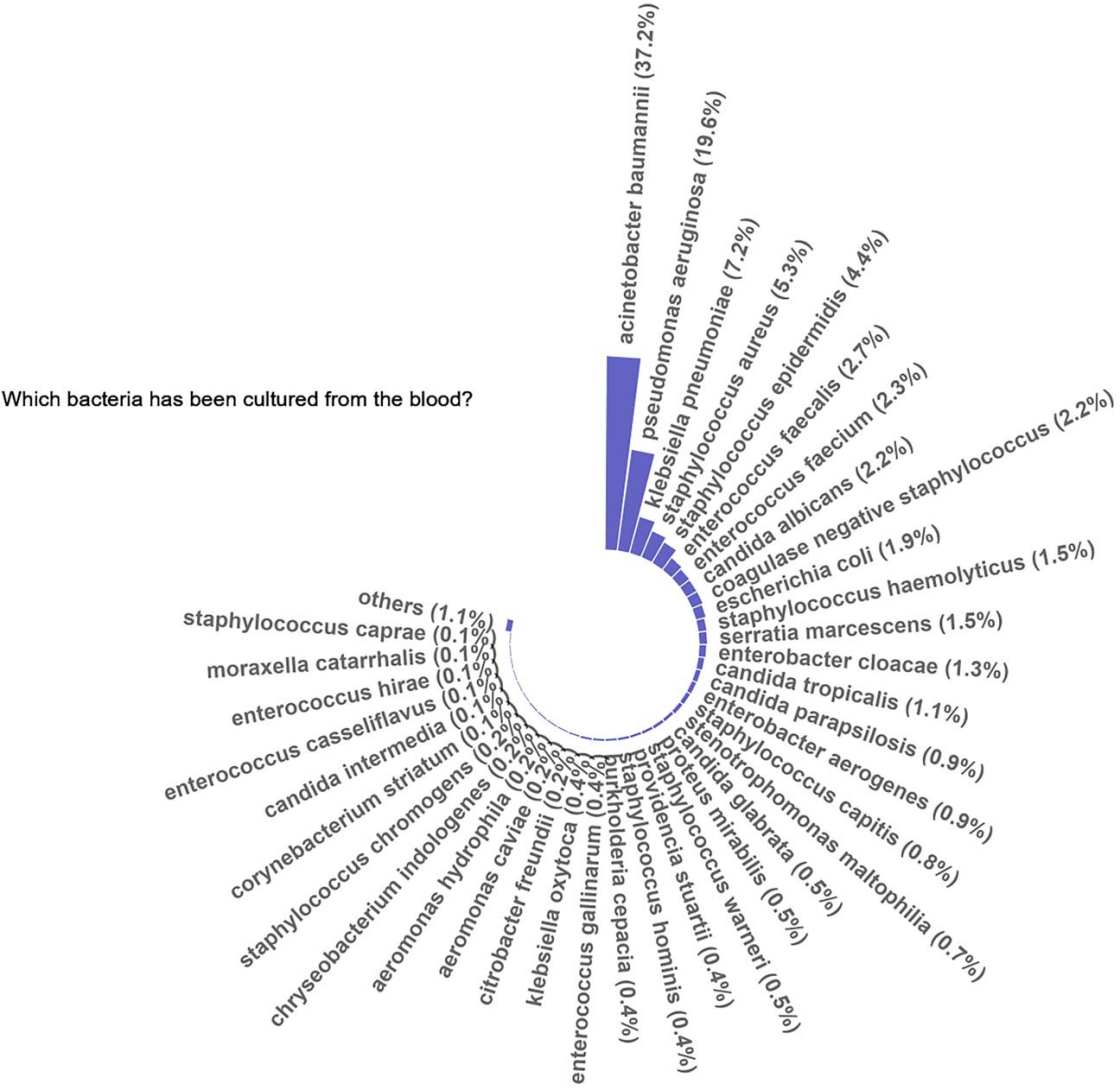
Frequency of bacteria in the blood culture-positive group

### Aetiology and prediction power for mortality

A total of 1646 bacterial cultures were obtained from 1643 patients with blood-positive sepsis. The most prevalent bacterial species identified was *Acinetobacter baumannii*, which was present in 37.2% of the cases. *Pseudomonas aeruginosa* was the second most common species, detected in 19.6% of the cases, followed by *Klebsiella pneumoniae* (7.2%) and *Staphylococcus aureus* (5.3%) (Figure 3). Among the identified bacterial species, those present at a frequency of higher than 2% were selected for the univariate analysis of mortality. These included *A. baumannii*, *P. aeruginosa*, *K. pneumoniae*, *S. aureus*, *Staphylococcus epidermidis*, *Enterococcus faecalis*, *Enterococcus faecium*, coagulase-negative *Staphylococcus* and *Candida albicans*. Four of these species, namely: *P. aeruginosa*, *S. aureus*, *S. epidermidis* and coagulase-negative *Staphylococcus*, were found to be statistically significant predictors of mortality. However, individuals with a positive blood culture for *P. aeruginosa* tend to have a more favourable prognosis than others (Table 4). Notably, the presence of multiple bacterial species in a single culture was not a significant predictor of mortality (*p* = 0.823).

**Table 4:** Univariable logistic analysis of mortality for bacteria

**Characteristic**	**OR (95% CI)**	***P* value**
*Acinetobacter baumannii*	0.867 (0.654, 1.149)	0.321
*Pseudomonas aeruginosa*	0.526 (0.388, 0.713)	**<0.001**
*Klebsiella pneumoniae*	1.006 (0.648, 1.580)	0.980
*Staphylococcus aureus*	2.236 (1.325, 3.958)	**0.002**
*Staphylococcus epidermidis*	2.218 (1.278, 4.062)	**0.004**
*Enterococcus faecalis*	1.349 (0.705, 2.722)	0.373
*Enterococcus faecium*	0.869 (0.447, 1.733)	0.683
Coagulase negative staphylococcus	4.684 (1.827, 15.88)	**<0.001**
*Candida albicans*	0.642 (0.278, 1.497)	0.299

*OR* odds ratio, *CI* confidence interval. The *p*-value was denoted in bold font to indicate statistical significance < 0.05

### Antibiotics resistance and prediction power for mortality

The resistance rates of *A. baumannii* to aztreonam, trimethoprim-sulfa, aminoglycoside and penicillin were 99.5, 93.8, 62.6 and 96.4%, respectively. Similarly, *P. aeruginosa* demonstrated resistance to aztreonam, colistin, trimethoprim-sulfa and penicillin at the rates of 66, 60, 65.3 and 57.6%, respectively. *K. pneumoniae* showed resistance to aztreonam, trimethoprim-sulfa, penicillin, cephalosporin, fluoroquinolone, carbapenem, tetracycline and carboxypenicillin at levels of 50%. *S. aureus* demonstrated resistance to penicillin, fluoroquinolone, erythromycin, telithromycin and lincosamide at levels of 50%. Likewise, *S. epidermidis* showed resistance to penicillin, fluoroquinolone, erythromycin, fusidic acid and lincosamide at levels 50% (Table 5). In *A. baumannii*, the odds ratio (OR) of carbapenem resistance for mortality was 4.403 (*p* = 0.024), whereas the OR of fluoroquinolone resistance for mortality was 3.144 (*p* = 0.024). In *K. pneumoniae*, resistance to carbapenem, penicillin, cefoxitin and cephalosporin was found to be statistically significant. However, the OR for cephalosporin resistance was 0.451, indicating that patients with resistance to this antimicrobial were better able to survive. Particularly, the only statistically significant resistance found in *S. epidermidis* was to tetracycline. (Table 6).

**Table 5:** Frequency of susceptibility of bacteria to antibiotics

	***Acinetobacter baumannii***	***Klebsiella pneumoniae***	***Pseudomonas aeruginosa***	***Staphylococcus aureus***	***Staphylococcus epidermidis***
Antibiotics	n = 659	n = 125	n = 360	n = 97	n = 77
Aztreonam					
Sensitive	3 (0.5%)	34 (34.0%)	104 (29.2%)		
Resistant	600 (99.5%)	66 (66.0%)	252 (70.8%)		
Colistin					
Sensitive	653 (99.1%)	8 (40.0%)	353 (98.1%)		
Resistant	6 (0.9%)	12 (60.0%)	7 (1.9%)		
Trimethoprim-sulfamethoxazole					
Sensitive	41 (6.2%)	43 (34.7%)	1 (0.3%)	58 (59.8%)	44 (57.1%)
Resistant	618 (93.8%)	81 (65.3%)	355 (99.7%)	39 (40.2%)	33 (42.9%)
Aminoglycoside					
Sensitive	237 (37.4%)	109 (87.9%)	171 (52.5%)	92 (94.8%)	74 (96.1%)
Resistant	397 (62.6%)	15 (12.1%)	155 (47.5%)	5 (5.2%)	3 (3.9%)
Penicillin					
Sensitive	24 (3.6%)	53 (42.4%)	99 (27.5%)	5 (5.2%)	12 (15.6%)
Resistant	635 (96.4%)	72 (57.6%)	261 (72.5%)	92 (94.8%)	65 (84.4%)
Cephalosporin					
Sensitive	19 (2.9%)	59 (47.2%)	120 (33.3%)		
Resistant	640 (97.1%)	66 (52.8%)	240 (66.7%)		
Fluoroquinolone					
Sensitive	21 (3.2%)	37 (30.1%)	85 (23.8%)	6 (6.2%)	34 (44.7%)
Resistant	635 (96.8%)	86 (69.9%)	272 (76.2%)	91 (93.8%)	42 (55.3%)
Carbapenem					
Sensitive	14 (2.1%)	92 (73.6%)	43 (11.9%)		
Resistant	645 (97.9%)	33 (26.4%)	317 (88.1%)		
Tetracycline					
Sensitive	610 (94.6%)	73 (83.0%)	4 (1.1%)	82 (85.4%)	75 (97.4%)
Resistant	35 (5.4%)	15 (17.0%)	351 (98.9%)	14 (14.6%)	2 (2.6%)
Carboxypenicillin					
Sensitive	15 (2.5%)		21 (6.2%)		
Resistant	582 (97.5%)		315 (93.8%)		
Cefoxitin					
Sensitive		69 (69.0%)			
Resistant		31 (31.0%)			
Erythromycin					
Sensitive				8 (8.2%)	33 (42.9%)
Resistant				89 (91.8%)	44 (57.1%)
Fusidic acid					
Sensitive				57 (62.0%)	18 (24.3%)
Resistant				35 (38.0%)	56 (75.7%)
Rifampin					
Sensitive				89 (91.8%)	55 (71.4%)
Resistant				8 (8.2%)	22 (28.6%)
Telithromycin					
Sensitive				38 (41.3%)	46 (62.2%)
Resistant				54 (58.7%)	28 (37.8%)
Lincosamide					
Sensitive				9 (9.5%)	37 (49.3%)
Resistant				86 (90.5%)	38 (50.7%)

**Table 6:** Univariable logistic analysis of mortality for antibiotic resistance in each bacterium

	***Acinetobacter baumannii***		***Klebsiella pneumoniae***		***Pseudomonas aeruginosa***		***Staphylococcus aureus***		***Staphylococcus epidermidis***	
**Antibiotics**	**OR (95% CI)**	***P* value**	**OR (95% CI)**	***P* value**	**OR (95% CI)**	***P* value**	**OR (95% CI)**	***P* value**	**OR (95% CI)**	***P* value**
Carbapenem		**0.024**		**0.007**		0.182				
Resistant	4.403 (1.188, 28.45)		3.027 (1.346, 7.023)		1.546 (0.816, 2.985)					
Fluoroquinolone		**0.024**		0.220		0.485		0.760		0.456
Resistant	3.144 (1.148, 11.02)		1.649 (0.745, 3.797)		1.190 (0.731, 1.941)		0.758 (0.139, 5.708)		0.650 (0.204, 2.032)	
Penicillin		0.081		**0.014**		0.836		0.547		0.700
Resistant	2.205 (0.911, 6.149)		2.533 (1.206, 5.499)		1.050 (0.660, 1.669)		0.560 (0.088, 4.436)		0.750 (0.191, 3.732)	
Aminoglycoside		0.082		0.924		0.516		0.122		0.079
Resistant	1.338 (0.96, 1.863)		0.948 (0.299, 2.817)		0.866 (0.560, 1.338)		4.250 (0.667, 33.77)		8.571 (0.770, 192.3)	
Colistin		0.185		>0.999		0.275				
Resistant	0.277 (0.014, 1.728)		1.000 (0.107, 7.949)		2.403 (0.511, 16.93)					
Carboxypenicillin		0.245				0.977				
Resistant	1.930 (0.651, 7.029)				1.013 (0.411, 2.469)					
Trimethoprim-sulfamethoxazole		0.347		0.196		0.227		0.947		0.625
Resistant	0.738 (0.391, 1.397)		1.657 (0.773, 3.662)		838 474 (0.000, NA)		1.031 (0.410, 2.536)		0.756 (0.232, 2.301)	
Cephalosporin		0.355		**0.030**		0.765				
Resistant	1.571 (0.613, 4.520)		0.451 (0.216, 0.927)		0.935 (0.603, 1.450)					
Tetracycline		0.863		0.890		0.271		0.893		**0.011**
Resistant	0.941 (0.460, 1.869)		1.087 (0.309, 3.437)		3.268 (0.414, 66.41)		1.091 (0.277, 3.643)		68 195 215 (0.000, NA)	
Cefoxitin				**0.043**						
Resistant			2.519 (1.029, 6.220)							
Fusidic acid								0.118		0.309
Resistant							0.448 (0.147, 1.218)		2.182 (0.518, 15.01)	
Rifampin								0.277		0.791
Resistant							0.346 (0.018, 2.084)		1.176 (0.331, 3.772)	
Telithromycin								0.601		0.420
Resistant							0.778 (0.304, 2.013)		0.600 (0.151, 2.025)	
Lincosamide								0.766		0.532
Resistant							1.278 (0.284, 8.995)		0.703 (0.224, 2.131)	
Erythromycin								0.850		0.625
Resistant							1.172 (0.250, 8.363)		1.324 (0.435, 4.319)	

*OR* odds ratio, *CI* confidence interval. The *p*-value was denoted in bold font to indicate statistical significance < 0.05

## Discussion

### Key findings

In this study, we examined routine biomarkers in burn patients with blood culture-positive and-negative sepsis because of the challenges associated with diagnosing and treating those without a positive blood culture. Among sepsis patients, the frequency of developing blood-negative sepsis was much higher than that of patients with blood-positive sepsis (Figure 2). Our findings revealed that pH, platelet count, bicarbonate and haematocrit were significant in both the positive and negative groups. However, lymphocyte, RDW and BUN were significant only in the positive group, and LD was significant only in the negative group. In addition, we identified *A. baumannii*, *P. aeruginosa* and *K. pneumonia* as common gram-negative bacterial species and *S. aureus* and *S. epidermidis* as common gram-positive bacterial species in burn patients with positive blood cultures. Furthermore, we found that carbapenem resistance was associated with an unfavourable prognosis in gram-negative bacteria, except in *P. aeruginosa*.

### Relationship to previous studies

Blood pH and bicarbonate are important physiological parameters that reflect the balance between acidity and alkalinity. Predicting pH and bicarbonate levels in burn patients with sepsis has been a topic of interest in the medical community as these markers are closely linked to mortality outcomes. [12,13]. A study reported that burn patients with sepsis with a significantly lower pH of 7.15 in the positive group and a pH.of 7.25 in the negative group, as well as a bicarbonate level of 17.5 mmol/L in both groups, had a significantly higher mortality rate compared to those with higher levels of these markers (Table S1 and S3). Thus, this finding suggests that acidosis, or an excess of acid in the blood, may be a marker of sepsis in burn patients. Platelets are small disk-shaped cells that are essential for blood clotting and wound healing. Evidence suggests that an abnormal platelet count, either too high or too low, is associated with an increased risk of sepsis and poor outcomes in burn patients. For example, Cato *et al*. [14] found that burn patients with sepsis had a significantly lower platelet count than those without sepsis, suggesting that thrombocytopenia or a deficiency of platelets in the blood may be a marker of sepsis in burn patients. Consistent with the aforementioned study, we found that clusters with a median platelet count of 75,000 in the positive group and 110,000 in the negative group were associated with higher mortality rates (Table S2). It is also well established that sepsis can lead to an overactive immune response, thereby decreasing the number of lymphocytes in the bloodstream. This decrease in lymphocyte count has been linked to an increased risk of mortality in patients with sepsis. In addition, apoptosis, the process of programmed cell death, plays a central role in this process by affecting the immune response at various levels. Research has shown that apoptosis-induced decrease in lymphocyte count may contribute to the increased mortality risk associated with sepsis [15]. Overall, monitoring lymphocyte counts may be a useful strategy for identifying sepsis in patients with positive blood cultures, according to our findings. Haematocrit, a measure of the volume of red blood cells in the blood, and RDW, a measure of the size of red blood cells, have been shown to be useful indicators of sepsis in various patient populations. Consistent with our results, numerous studies have shown that low haematocrit and high RDW are correlated with unfavourable outcomes in patients with sepsis [16–18]. Serum LD levels are probably associated with mortality in patients with sepsis, as studies have suggested that this relationship may be due to the fact that sepsis is associated with tissue damage and inflammation, and elevated LD values can be a marker of this damage. [19,20]. In the present study, LD also showed a higher level in the high-risk cluster in the negative group. *A. baumannii*, *P. aeruginosa*, *K. pneumoniae*, *S. aureus* and *S. epidermidis* are common bacterial pathogens that can cause severe infections in patients with burns and sepsis. Since these infections can lead to high morbidity and mortality, effective prevention and treatment strategies are critical for improving the outcomes in this affected population, as these bacteria are frequently isolated from the blood of burn patients and are often resistant to multiple antimicrobial agents, making them difficult to treat. [21]. Although the frequencies varied slightly, similar strains were observed in the present study. The treatment of these infections typically involves the use of antibiotics, although the choice of antimicrobials should be guided by the results of susceptibility testing and should consider the patient’s underlying health conditions and potential drug–drug interactions. In the present study, carbapenem resistance in gram-negative bacteria and tetracycline resistance in gram-positive bacteria are the major challenges in managing burn sepsis. Gram-negative bacteria have a thin peptidoglycan layer and an outer membrane that makes them resistant to many antimicrobial agents. On the other hand, gram-positive bacteria have a thick peptidoglycan layer in their cell walls, which makes them resistant to many antimicrobial agents. Carbapenems, a class of beta-lactam antibiotics, are often used as a last-resort treatment for infections caused by gram-negative bacteria owing to their ability to penetrate the outer membrane and inhibit cell wall synthesis. However, the widespread use of carbapenems has led to the emergence of carbapenem-resistant gram-negative bacteria, and tetracyclines, a class of antibiotics that inhibit protein synthesis, are commonly used to treat infections caused by gram-positive bacteria [22]. The emergence of tetracycline-resistant gram-positive bacteria has also become a major concern in recent years because these bacteria are resistant to most of the available antimicrobial agents. [23].

### Strengths and limitations

This study is the first to utilize longitudinal data and *k*-means clustering algorithms to categorize and identify significant biomarkers in sepsis with positive or negative blood cultures in burn patients. By utilizing a deep learning mechanism and the largest available dataset, we were able to identify meaningful biomarkers within the heterogeneity of burn patients without the influence of human selection bias. However, it should be noted that this study was conducted at a single centre, and, therefore, may be subject to geographical bias, limiting the generalizability of our findings to other populations. Additionally, the clinical implications of positive or negative blood cultures may not be immediately clear. Nevertheless, we believe that by identifying the differences and laboratory markers associated with these cultures, further investigations into the complex nature of sepsis in burn patients could be guided. This, in turn, could lead to the development of more accurate predictive models and more targeted and effective treatment strategies, while also validating our study design for other institutions.

## Conclusions

We found routine biomarkers of sepsis in burn patients, including pH, platelet count, bicarbonate level and haematocrit level, to be statistically significant in both blood culture groups. Only LD was significant in the blood-negative group, while RDW, BUN and lymphocyte count were significant in the positive group. Gram-negative bacteria, including *A. baumannii*, *K. pneumoniae* and *P. aeruginosa*, were the most common causes of sepsis in burn patients, with resistance to carbapenems associated with unfavourable outcomes. Our study provides insights into the treatment of burn patients, though further research is necessary to explore issues such as multi-resistance and the presence of bacteria in locations other than the blood.

## Supplementary Material

suppMaterial_tkad031

## Data Availability

The data that support the findings of this study are available from the corresponding author upon reasonable request.
