# Supplementary material for: The clinical differentiation of blood culture-positive and -negative sepsis in burn patients: a retrospective cohort study
Source: Burns Trauma. 2023 Dec 18;11:tkad031. doi: 10.1093/burnst/tkad031 (PMC10729782; doi:10.1093/burnst/tkad031)
Supplement: suppMaterial_tkad031 [file suppmaterial_tkad031.docx]

**Supplementary material**

**The Clinical Differentiation of Blood Culture-Positive and Negative Sepsis in Burn Patients: A retrospective cohort study**

Jaechul Yoon^1,2,3,^, Dohern Kym^1,3^, Jun Hur^1,3*^, Jongsoo Park^1,3^, Myongjin Kim^1,3^, Yong-Suk Cho^1,3^, Wook Chun^1,3^, and Dogeon Yoon^3^

^1^Department of Surgery and Critical Care, Burn Center, Hangang Sacred Heart Hospital, Hallym University Medical Center, 12, Beodeunaru-ro 7-gil, Youngdeungpo-gu, Seoul, Korea, 07247

^2^ Graduate school of Medicine, Kanwon National University, Chuncheon, Republic of Korea

^3^Burn Institutes, Hangang Sacred Heart Hospital, Hallym University Medical Center, 12, Beodeunaru-ro 7-gil, Youngdeungpo-gu, Seoul, Korea, 07247

*Corresponding authors

Department of Surgery and Critical Care, Burn Center, Hangang Sacred Heart Hospital, College of Medicine, Hallym University 12, Beodeunaru-ro 7-gil, Youngdeungpo-gu, Seoul, Korea, 07247

Tel. 82-2-2639-5446, Fax. 82-2-2678-4386, E-mail: [hammerj@hallym.or.kr](mailto:hammerj@hallym.or.kr)

Contents

[STROBE Statement—Checklist of items that should be included in reports of cohort studies 4](#_Toc122500176)

[Fig S1. Longitudinal pH Values Classified by kmlShape Package: Individual and Mean Trajectories 7](#_Toc122500177)

[Table S1. Characteristics and pH Values Changes Over Time for Each Cluster in the Blood Culture-Positive/Negative Group 7](#_Toc122500178)

[Fig S2. Longitudinal Platelet Counts Classified by kmlShape Package: Individual and Mean Trajectories 9](#_Toc122500179)

[Table S2. Characteristics and Platelet Counts Changes Over Time for Each Cluster in the Blood Culture-Positive/Negative Group 9](#_Toc122500180)

[Fig S3. Longitudinal Bicarbonate levels Classified by kmlShape Package: Individual and Mean Trajectories 11](#_Toc122500181)

[Table S3. Characteristics and Bicarbonate levels Changes Over Time for Each Cluster in the Blood Culture-Positive/Negative Group 11](#_Toc122500182)

[Fig S4. Longitudinal Haematocrit levels Classified by kmlShape Package: Individual and Mean Trajectories 13](#_Toc122500183)

[Table S4. Characteristics and Haematocrit levels Changes Over Time for Each Cluster in the Blood Culture-Positive/Negative Group 13](#_Toc122500184)

[Fig S5. Longitudinal RDW levels Classified by kmlShape Package: Individual and Mean Trajectories 15](#_Toc122500185)

[Table S5. Characteristics and RDW levels Changes Over Time for Each Cluster in the Blood Culture-Positive/Negative Group 15](#_Toc122500186)

[Fig S6. Longitudinal Lymphocyte Counts Classified by kmlShape Package: Individual and Mean Trajectories 17](#_Toc122500187)

[Table S6. Characteristics and Lymphocyte Counts Changes Over Time for Each Cluster in the Blood Culture-Positive/Negative Group 17](#_Toc122500188)

[Fig S7. Longitudinal BUN levels Classified by kmlShape Package: Individual and Mean Trajectories 19](#_Toc122500189)

[Table S7. Characteristics and BUN levels Changes Over Time for Each Cluster in the Blood Culture-Positive/Negative Group 19](#_Toc122500190)

[Fig S8. Longitudinal LD levels Classified by kmlShape Package: Individual and Mean Trajectories 21](#_Toc122500191)

[Table S8. Characteristics and LD levels Changes Over Time for Each Cluster in the Blood Culture-Positive/Negative Group 21](#_Toc122500192)

# STROBE Statement—Checklist of items that should be included in reports of cohort studies

|  | | Item No | | Recommendation | Checked | |  |
| --- | --- | --- | --- | --- | --- | --- | --- |
| **Title and abstract** | | 1 | | (*a*) Indicate the study’s design with a commonly used term in the title or the abstract | Yes | |  |
|  |  |  |  | (*b*) Provide in the abstract an informative and balanced summary of what was done and what was found | Yes | |  |
| Introduction | | | | | | |  |
| Background/rationale | | 2 | | Explain the scientific background and rationale for the investigation being reported | Yes | |  |
| Objectives | | 3 | | State specific objectives, including any prespecified hypotheses | Yes | |  |
| Methods | | | | | | |  |
| Study design | | 4 | | Present key elements of study design early in the paper | Yes | |  |
| Setting | | 5 | | Describe the setting, locations, and relevant dates, including periods of recruitment, exposure, follow-up, and data collection | Yes | |  |
| Participants | | 6 | | (*a*) Give the eligibility criteria, and the sources and methods of selection of participants. Describe methods of follow-up | Yes | |  |
|  |  |  |  | (*b*) For matched studies, give matching criteria and number of exposed and unexposed | Yes | |  |
| Variables | | 7 | | Clearly define all outcomes, exposures, predictors, potential confounders, and effect modifiers. Give diagnostic criteria, if applicable | Yes | |  |
| Data sources/ measurement | | 8* | | For each variable of interest, give sources of data and details of methods of assessment (measurement). Describe comparability of assessment methods if there is more than one group | Yes | |  |
| Bias | | 9 | | Describe any efforts to address potential sources of bias | Yes | |  |
| Study size | | 10 | | Explain how the study size was arrived at | No | |  |
| Quantitative variables | | 11 | | Explain how quantitative variables were handled in the analyses. If applicable, describe which groupings were chosen and why | Yes | |  |
| Statistical methods | | 12 | | (*a*) Describe all statistical methods, including those used to control for confounding | Yes | |  |
|  |  |  |  | (*b*) Describe any methods used to examine subgroups and interactions | Yes | |  |
|  |  |  |  | (*c*) Explain how missing data were addressed | Yes | |  |
|  |  |  |  | (*d*) If applicable, explain how loss to follow-up was addressed | NA | |  |
|  |  |  |  | (*e*) Describe any sensitivity analyses | NA | |  |
| Results | | | | |  | |  |
| Participants | | 13* | | (a) Report numbers of individuals at each stage of study—eg numbers potentially eligible, examined for eligibility, confirmed eligible, included in the study, completing follow-up, and analysed | Yes | |  |
|  |  |  |  | (b) Give reasons for non-participation at each stage | NA | |  |
|  |  |  |  | (c) Consider use of a flow diagram | Yes | |  |
| Descriptive data | | 14* | | (a) Give characteristics of study participants (eg demographic, clinical, social) and information on exposures and potential confounders | Yes | |  |
|  |  |  |  | (b) Indicate number of participants with missing data for each variable of interest | Yes | |  |
|  |  |  |  | (c) Summarise follow-up time (eg, average and total amount) | NA | |  |
| Outcome data | | 15* | | Report numbers of outcome events or summary measures over time | Yes | |  |
| Main results | 16 | | (*a*) Give unadjusted estimates and, if applicable, confounder-adjusted estimates and their precision (eg, 95% confidence interval). Make clear which confounders were adjusted for and why they were included | | | Yes | |
|  |  |  | (*b*) Report category boundaries when continuous variables were categorized | | | Yes | |
|  |  |  | (*c*) If relevant, consider translating estimates of relative risk into absolute risk for a meaningful time period | | | Yes | |
| Other analyses | 17 | | Report other analyses done—eg analyses of subgroups and interactions, and sensitivity analyses | | | Yes | |
| Discussion | | | | | | | |
| Key results | 18 | | Summarise key results with reference to study objectives | | | Yes | |
| Limitations | 19 | | Discuss limitations of the study, taking into account sources of potential bias or imprecision. Discuss both direction and magnitude of any potential bias | | | Yes | |
| Interpretation | 20 | | Give a cautious overall interpretation of results considering objectives, limitations, multiplicity of analyses, results from similar studies, and other relevant evidence | | | Yes | |
| Generalisability | 21 | | Discuss the generalisability (external validity) of the study results | | | Yes | |
| Other information | | | | | | | |
| Funding | 22 | | Give the source of funding and the role of the funders for the present study and, if applicable, for the original study on which the present article is based | | | Yes | |

*Give information separately for exposed and unexposed groups.

**Note:** An Explanation and Elaboration article discusses each checklist item and gives methodological background and published examples of transparent reporting. The STROBE checklist is best used in conjunction with this article (freely available on the Web sites of PLoS Medicine at http://www.plosmedicine.org/, Annals of Internal Medicine at http://www.annals.org/, and Epidemiology at http://www.epidem.com/). Information on the STROBE Initiative is available at http://www.strobe-statement.org.

# Longitudinal profiles of predictors

The longitudinal profiles were divided into clusters using k-means clustering to ensure that they were not physician-biased. We examined four biomarkers, namely: pH, platelet count, RDW, and lymphocyte count in relation to the levels of each biomarker in the flow. Notably, the longitudinal profiles of the pH biomarkers showed a declining trend over time in cluster C, which had the highest mortality rate (97.8%). In the positive group, the median pH level ranged from 7.42 on day 1 to 7.43 on day 7, whereas in the negative group, the median pH level ranged from 7.42 on day 1 to 7.44 on day 7. (Table S1)

The longitudinal profiles of the platelet biomarker showed an incline, but the incline was narrower than that of the other clusters over time in cluster C, which had the highest mortality rate. In the positive group, the median platelet count ranged from 363 on day 1 to 529 on day 7; however, in the negative group, the median platelet count ranged from 359 on day 1 to 538 on day 7. (Table S2)

Notably, the patterns of RDW and lymphocyte biomarkers were similar and flat in all clusters. In the positive group, the mean-median lymphocyte level in cluster C, which had the highest mortality rate (67.4 %), was 0.43. In contrast, in the negative group, the mean-median lymphocyte level in cluster C, which had the highest mortality rate (33.4 %), was 0.57. (Table S3) In addition, in the positive group, the mean-median RDW in cluster C, which had the highest mortality rate of 71.2%, was 16.30; however, in the negative group, the mean-median RDW in cluster C, which had the highest mortality rate (45.9 %), was 16.10. (Table S4)

# Fig S1. Longitudinal pH Values Displayed in Raincloud Plots Categorized by kmlShape Package. (a. Blood Positive, b. Blood Negative)


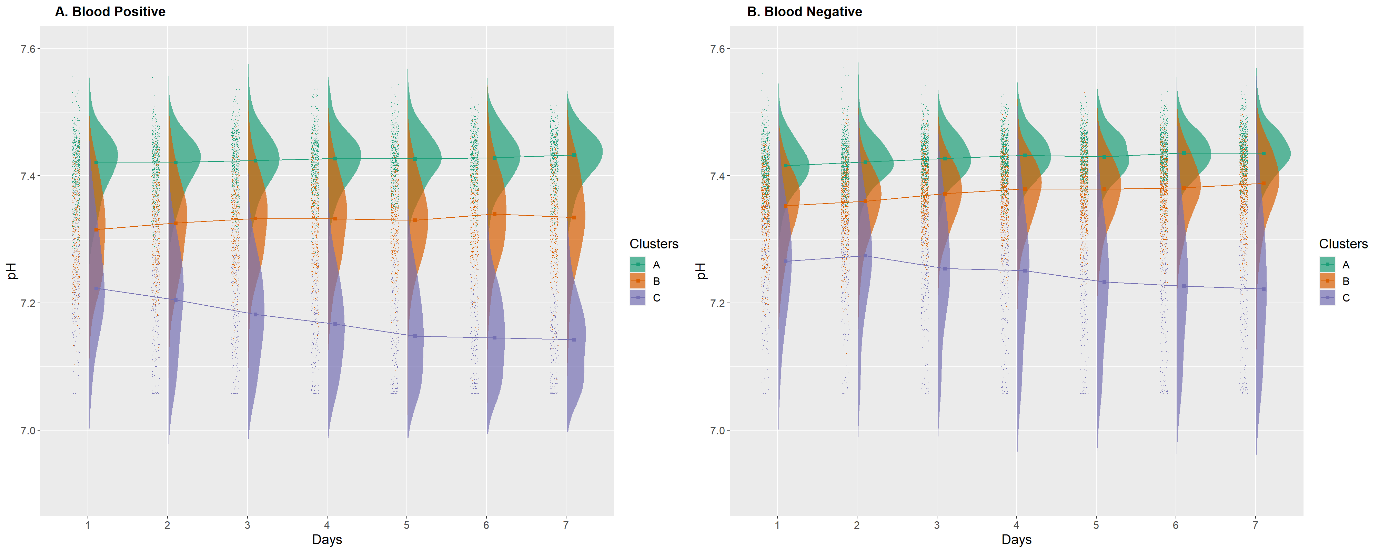


# Table S1. Characteristics and pH Values Changes Over Time for Each Cluster in the Blood Culture-Positive/Negative Group

|  |  | Blood Positive | | | | Blood Negative | | | |
| --- | --- | --- | --- | --- | --- | --- | --- | --- | --- |
| Group | Variables | A, N = 383 (46.1%) | B, N = 308 (37.1%) | C, N = 139 (16.7%) | p-value | A, N = 493 (41.2%) | B, N = 533 (44.6%) | C, N = 170 (14.2%) | p-value |
| Demographics | Mortality | 28 (7.3%) | 146 (47.4%) | 136 (97.8%) | <0.001 | 18 (3.7%) | 96 (18.0%) | 123 (72.4%) | <0.001 |
|  | Patient Age |  |  |  | 0.283 |  |  |  | 0.008 |
|  | Median [IQR] | 52 [42, 62] | 53 [42, 64] | 51 [42, 64] |  | 52 [43, 64] | 51 [40, 62] | 56 [47, 68] |  |
|  | Sex |  |  |  | 0.083 |  |  |  | 0.277 |
|  | Male | 290 (75.7%) | 254 (82.5%) | 112 (80.6%) |  | 377 (76.5%) | 412 (77.3%) | 140 (82.4%) |  |
|  | Female | 93 (24.3%) | 54 (17.5%) | 27 (19.4%) |  | 116 (23.5%) | 121 (22.7%) | 30 (17.6%) |  |
|  | TBSA |  |  |  | <0.001 |  |  |  | <0.001 |
|  | Median [IQR] | 35 [24, 49] | 48 [33, 64] | 70 [45, 85] |  | 24 [13, 35] | 28 [17, 44] | 54 [30, 75] |  |
|  | Inhalation | 155 (40.5%) | 160 (51.9%) | 86 (61.9%) | <0.001 | 139 (28.2%) | 239 (44.8%) | 108 (63.5%) | <0.001 |
|  | LOICU |  |  |  | <0.001 |  |  |  | <0.001 |
|  | Median [IQR] | 29 [21, 44] | 34 [22, 50] | 12 [10, 16] |  | 19 [9, 30] | 25 [13, 39] | 16 [8, 34] |  |
| pH value | Day 1 |  |  |  | <0.001 |  |  |  | <0.001 |
|  | Median [IQR] | 7.42 [7.38, 7.45] | 7.32 [7.25, 7.37] | 7.22 [7.18, 7.29] |  | 7.42 [7.39, 7.44] | 7.35 [7.31, 7.39] | 7.27 [7.21, 7.32] |  |
|  | Day 2 |  |  |  | <0.001 |  |  |  | <0.001 |
|  | Median [IQR] | 7.42 [7.39, 7.45] | 7.33 [7.27, 7.36] | 7.20 [7.13, 7.26] |  | 7.42 [7.40, 7.45] | 7.36 [7.32, 7.39] | 7.27 [7.22, 7.32] |  |
|  | Day 3 |  |  |  | <0.001 |  |  |  | <0.001 |
|  | Median [IQR] | 7.42 [7.39, 7.45] | 7.33 [7.28, 7.37] | 7.18 [7.13, 7.23] |  | 7.43 [7.40, 7.45] | 7.37 [7.34, 7.40] | 7.25 [7.20, 7.32] |  |
|  | Day 4 |  |  |  | <0.001 |  |  |  | <0.001 |
|  | Median [IQR] | 7.43 [7.40, 7.45] | 7.33 [7.28, 7.38] | 7.17 [7.12, 7.22] |  | 7.43 [7.41, 7.46] | 7.38 [7.34, 7.41] | 7.25 [7.17, 7.30] |  |
|  | Day 5 |  |  |  | <0.001 |  |  |  | <0.001 |
|  | Median [IQR] | 7.43 [7.40, 7.45] | 7.33 [7.28, 7.37] | 7.15 [7.08, 7.20] |  | 7.43 [7.41, 7.46] | 7.38 [7.34, 7.41] | 7.23 [7.16, 7.29] |  |
|  | Day 6 |  |  |  | <0.001 |  |  |  | <0.001 |
|  | Median [IQR] | 7.43 [7.40, 7.45] | 7.34 [7.29, 7.38] | 7.15 [7.09, 7.20] |  | 7.44 [7.41, 7.46] | 7.38 [7.35, 7.41] | 7.23 [7.16, 7.29] |  |
|  | Day 7 |  |  |  | <0.001 |  |  |  | <0.001 |
|  | Median [IQR] | 7.43 [7.41, 7.46] | 7.33 [7.28, 7.38] | 7.14 [7.08, 7.18] |  | 7.44 [7.41, 7.46] | 7.39 [7.35, 7.42] | 7.22 [7.15, 7.30] |  |
| Mean of overall median value | pH |  |  |  | <0.001 |  |  |  | <0.001 |
|  | Median [IQR] | 7.43 [7.40, 7.45] | 7.33 [7.28, 7.37] | 7.17 [7.12, 7.23] |  | 7.43 [7.40, 7.45] | 7.37 [7.34, 7.41] | 7.25 [7.18, 7.31] |  |

# Fig S2. Longitudinal Platelet Counts Displayed in Raincloud Plots Categorized by kmlShape Package. (a. Blood Positive, b. Blood Negative)


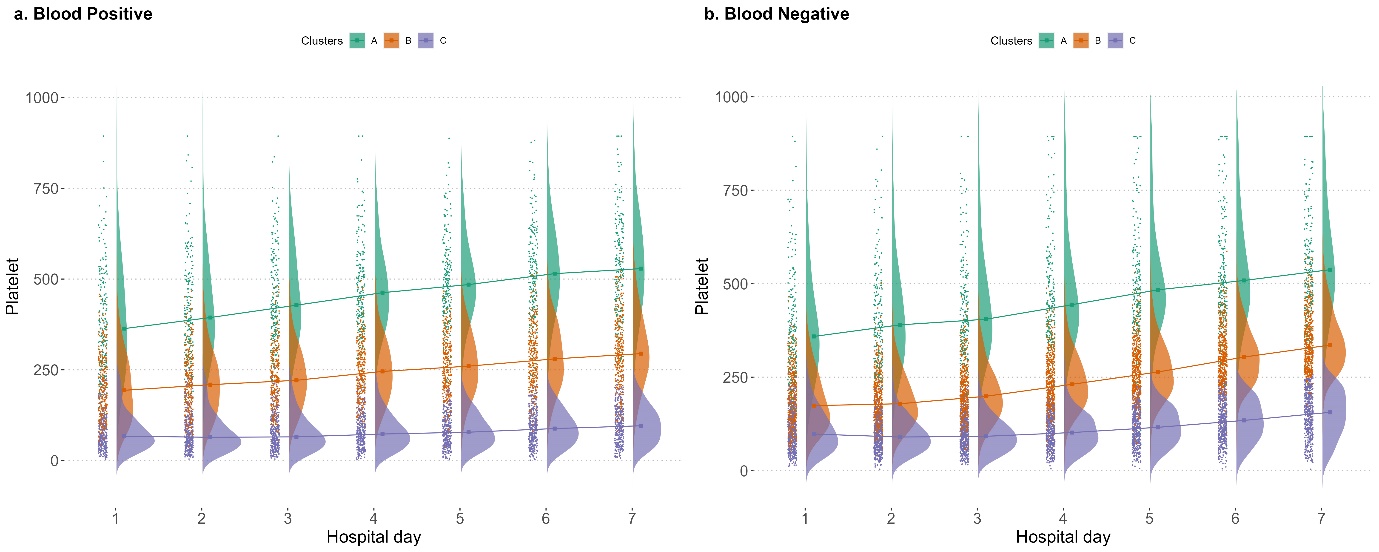


# Table S2. Characteristics and Platelet Counts Changes Over Time for Each Cluster in the Blood Culture-Positive/Negative Group

|  |  | Blood Positive | | | | Blood Negative | | | |
| --- | --- | --- | --- | --- | --- | --- | --- | --- | --- |
| Group | Variables | A, N = 194 (23.4%) | B, N = 306 (36.9%) | C, N = 330 (39.8%) | p-value | A, N = 191 (15.9%) | B, N = 523 (43.7%) | C, N = 484 (40.4%) | p-value |
| Demographics | Mortality | 5 (2.6%) | 60 (19.6%) | 245 (74.2%) | <0.001 | 8 (4.2%) | 26 (5.0%) | 203 (41.9%) | <0.001 |
|  | Patient Age |  |  |  | <0.001 |  |  |  | <0.001 |
|  | Median [IQR] | 48 [37, 56] | 53 [44, 64] | 54 [42, 66] |  | 49 [40, 58] | 50 [41, 60] | 56 [46, 69] |  |
|  | Sex |  |  |  | 0.385 |  |  |  | 0.835 |
|  | Male | 159 (82.0%) | 235 (76.8%) | 262 (79.4%) |  | 146 (76.4%) | 410 (78.4%) | 375 (77.5%) |  |
|  | Female | 35 (18.0%) | 71 (23.2%) | 68 (20.6%) |  | 45 (23.6%) | 113 (21.6%) | 109 (22.5%) |  |
|  | TBSA |  |  |  | <0.001 |  |  |  | <0.001 |
|  | Median [IQR] | 38 [26, 50] | 40 [28, 56] | 58 [34, 78] |  | 27 [16, 40] | 25 [14, 36] | 37 [20, 60] |  |
|  | Inhalation | 94 (48.5%) | 136 (44.4%) | 171 (51.8%) | 0.170 | 53 (27.7%) | 189 (36.1%) | 244 (50.4%) | <0.001 |
|  | LOICU |  |  |  | <0.001 |  |  |  | <0.001 |
|  | Median [IQR] | 30 [22, 41] | 34 [22, 49] | 20 [13, 34] |  | 24 [14, 34] | 18 [9, 32] | 21 [10, 39] |  |
| Platelet value | Day 1 |  |  |  | <0.001 |  |  |  | <0.001 |
|  | Median [IQR] | 363 [268, 490] | 194 [127, 270] | 67 [44, 108] |  | 359 [286, 461] | 174 [129, 236] | 98 [64, 143] |  |
|  | Day 2 |  |  |  | <0.001 |  |  |  | <0.001 |
|  | Median [IQR] | 394 [322, 497] | 209 [150, 269] | 64 [40, 104] |  | 390 [308, 490] | 179 [137, 234] | 90 [61, 123] |  |
|  | Day 3 |  |  |  | <0.001 |  |  |  | <0.001 |
|  | Median [IQR] | 428 [359, 543] | 221 [166, 287] | 65 [41, 107] |  | 405 [344, 520] | 199 [154, 250] | 92 [63, 123] |  |
|  | Day 4 |  |  |  | <0.001 |  |  |  | <0.001 |
|  | Median [IQR] | 462 [397, 539] | 246 [188, 306] | 73 [44, 107] |  | 444 [376, 552] | 231 [183, 287] | 102 [70, 140] |  |
|  | Day 5 |  |  |  | <0.001 |  |  |  | <0.001 |
|  | Median [IQR] | 485 [422, 561] | 260 [203, 323] | 78 [46, 115] |  | 484 [418, 576] | 264 [220, 320] | 116 [80, 157] |  |
|  | Day 6 |  |  |  | <0.001 |  |  |  | <0.001 |
|  | Median [IQR] | 515 [440, 606] | 280 [219, 350] | 88 [54, 123] |  | 509 [441, 615] | 304 [252, 356] | 134 [93, 179] |  |
|  | Day 7 |  |  |  | <0.001 |  |  |  | <0.001 |
|  | Median [IQR] | 529 [444, 630] | 295 [233, 371] | 96 [57, 134] |  | 538 [459, 650] | 336 [289, 394] | 156 [108, 204] |  |
| Mean of overall median value | Platelet |  |  |  | <0.001 |  |  |  | <0.001 |
|  | Median [IQR] | 461 [373, 557] | 244 [179, 312] | 75 [46, 115] |  | 453 [369, 556] | 243 [179, 312] | 110 [73, 154] |  |

# Fig S3. Longitudinal Lymphocyte Counts Displayed in Raincloud Plots Categorized by kmlShape Package. (a. Blood Positive, b. Blood Negative)


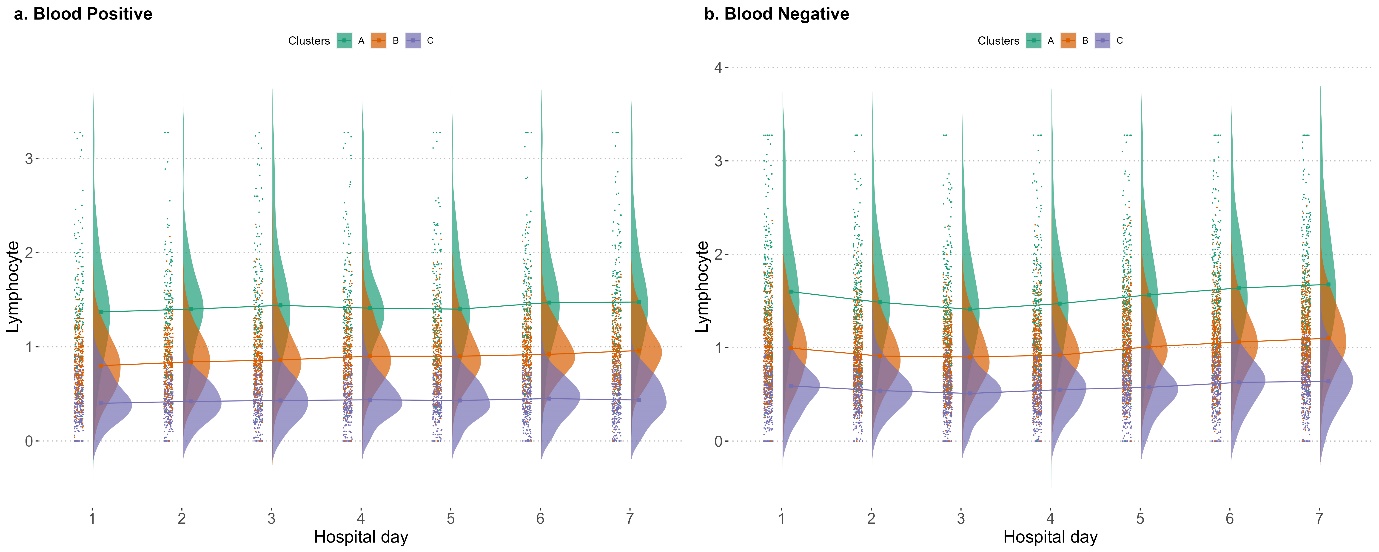


# Table S3. Characteristics and Lymphocyte Counts Changes Over Time for Each Cluster in the Blood Culture-Positive/Negative Group

|  |  | Blood Positive | | | | Blood Negative | | | |
| --- | --- | --- | --- | --- | --- | --- | --- | --- | --- |
| Group | Variables | A, N = 163 (19.6%) | B, N = 379 (45.7%) | C, N = 288 (34.7%) | p-value | A, N = 241 (20.1%) | B, N = 556 (46.4%) | C, N = 401 (33.5%) | p-value |
| Demographics | Mortality | 26 (16.0%) | 90 (23.7%) | 194 (67.4%) | <0.001 | 19 (7.9%) | 84 (15.1%) | 134 (33.4%) | <0.001 |
|  | Patient Age |  |  |  | <0.001 |  |  |  | <0.001 |
|  | Median [IQR] | 50 [42, 61] | 51 [40, 61] | 55 [45, 67] |  | 51 [43, 60] | 50 [41, 62] | 56 [46, 70] |  |
|  | Sex |  |  |  | 0.392 |  |  |  | 0.103 |
|  | Male | 132 (81.0%) | 304 (80.2%) | 220 (76.4%) |  | 178 (73.9%) | 446 (80.2%) | 307 (76.6%) |  |
|  | Female | 31 (19.0%) | 75 (19.8%) | 68 (23.6%) |  | 63 (26.1%) | 110 (19.8%) | 94 (23.4%) |  |
|  | TBSA |  |  |  | <0.001 |  |  |  | <0.001 |
|  | Median [IQR] | 36 [24, 46] | 40 [28, 60] | 54 [34, 72] |  | 24 [12, 33] | 27 [16, 42] | 33 [20, 52] |  |
|  | Inhalation | 70 (42.9%) | 187 (49.3%) | 144 (50.0%) | 0.313 | 83 (34.4%) | 226 (40.6%) | 177 (44.1%) | 0.049 |
|  | LOICU |  |  |  | <0.001 |  |  |  | 0.006 |
|  | Median [IQR] | 26 [18, 38] | 32 [21, 46] | 22 [13, 38] |  | 17 [9, 31] | 21 [11, 35] | 23 [11, 38] |  |
| Lymphocyte value | Day 1 |  |  |  | <0.001 |  |  |  | <0.001 |
|  | Median [IQR] | 1.37 [1.02, 1.70] | 0.80 [0.59, 1.00] | 0.40 [0.29, 0.60] |  | 1.60 [1.30, 1.98] | 1.00 [0.72, 1.23] | 0.59 [0.40, 0.73] |  |
|  | Day 2 |  |  |  | <0.001 |  |  |  | <0.001 |
|  | Median [IQR] | 1.40 [1.11, 1.68] | 0.84 [0.63, 1.06] | 0.42 [0.30, 0.60] |  | 1.49 [1.19, 1.88] | 0.91 [0.70, 1.15] | 0.54 [0.40, 0.70] |  |
|  | Day 3 |  |  |  | <0.001 |  |  |  | <0.001 |
|  | Median [IQR] | 1.44 [1.14, 1.78] | 0.86 [0.67, 1.08] | 0.43 [0.27, 0.60] |  | 1.41 [1.12, 1.84] | 0.90 [0.70, 1.15] | 0.51 [0.37, 0.67] |  |
|  | Day 4 |  |  |  | <0.001 |  |  |  | <0.001 |
|  | Median [IQR] | 1.41 [1.20, 1.73] | 0.90 [0.71, 1.12] | 0.44 [0.31, 0.60] |  | 1.47 [1.10, 1.84] | 0.92 [0.72, 1.15] | 0.55 [0.36, 0.70] |  |
|  | Day 5 |  |  |  | <0.001 |  |  |  | <0.001 |
|  | Median [IQR] | 1.40 [1.13, 1.79] | 0.90 [0.73, 1.13] | 0.43 [0.30, 0.60] |  | 1.57 [1.25, 1.95] | 1.01 [0.81, 1.28] | 0.58 [0.41, 0.73] |  |
|  | Day 6 |  |  |  | <0.001 |  |  |  | <0.001 |
|  | Median [IQR] | 1.47 [1.17, 1.79] | 0.92 [0.79, 1.12] | 0.45 [0.30, 0.60] |  | 1.64 [1.36, 2.05] | 1.06 [0.86, 1.30] | 0.63 [0.42, 0.79] |  |
|  | Day 7 |  |  |  | <0.001 |  |  |  | <0.001 |
|  | Median [IQR] | 1.48 [1.14, 1.80] | 0.96 [0.77, 1.16] | 0.44 [0.30, 0.60] |  | 1.68 [1.31, 2.10] | 1.10 [0.90, 1.35] | 0.64 [0.44, 0.81] |  |
| Mean of overall median value | Lymphocyte |  |  |  | <0.001 |  |  |  | <0.001 |
|  | Median [IQR] | 1.41 [1.14, 1.76] | 0.90 [0.70, 1.10] | 0.43 [0.30, 0.60] |  | 1.54 [1.24, 1.95] | 0.99 [0.77, 1.23] | 0.57 [0.40, 0.73] |  |

# Fig S4. Longitudinal RDW levels Displayed in Raincloud Plots Categorized by kmlShape Package. (a. Blood Positive, b. Blood Negative)


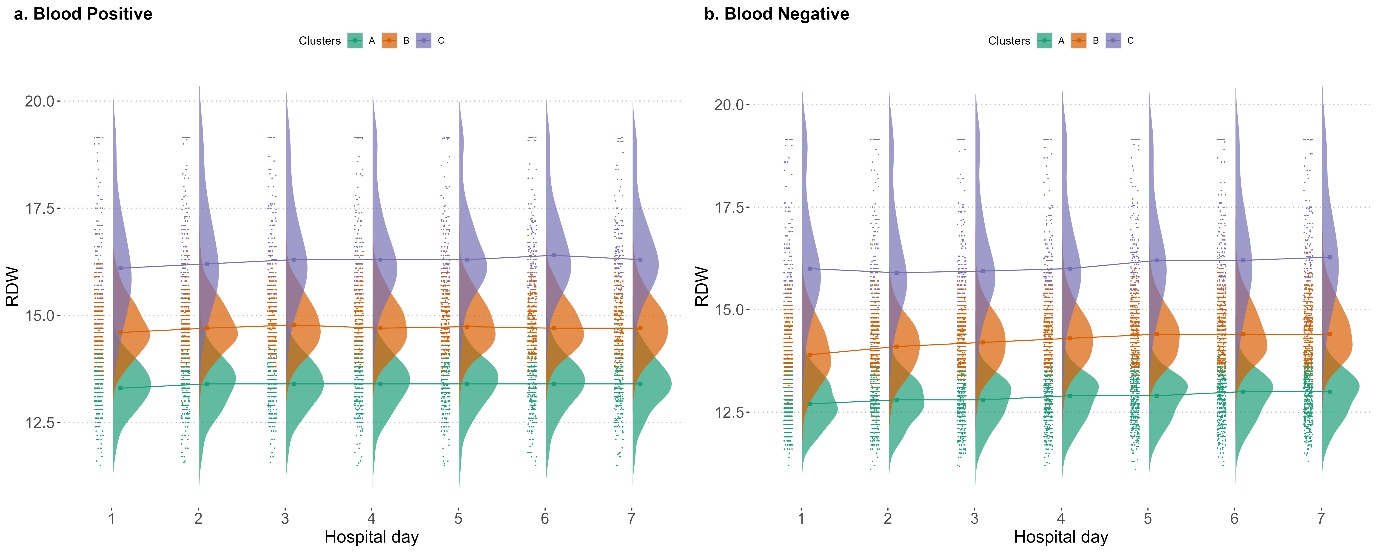


# Table S4. Characteristics and RDW levels Changes Over Time for Each Cluster in the Blood Culture-Positive/Negative Group

|  |  | Blood Positive | | | | Blood Negative | | | |
| --- | --- | --- | --- | --- | --- | --- | --- | --- | --- |
| Group | Variables | A, N = 291 (35.1%) | B, N = 400 (48.2%) | C, N = 139 (16.7%) | p-value | A, N = 502 (41.9%) | B, N = 550 (45.9%) | C, N = 146 (12.2%) | p-value |
| Demographics | Mortality | 22 (7.6%) | 189 (47.2%) | 99 (71.2%) | <0.001 | 17 (3.4%) | 153 (27.8%) | 67 (45.9%) | <0.001 |
|  | Patient Age |  |  |  | <0.001 |  |  |  | <0.001 |
|  | Median [IQR] | 50 [41, 60] | 52 [42, 63] | 57 [45, 66] |  | 49 [39, 59] | 54 [44, 67] | 59 [48, 71] |  |
|  | Sex |  |  |  | 0.447 |  |  |  | 0.002 |
|  | Male | 237 (81.4%) | 311 (77.8%) | 108 (77.7%) |  | 414 (82.5%) | 409 (74.4%) | 108 (74.0%) |  |
|  | Female | 54 (18.6%) | 89 (22.2%) | 31 (22.3%) |  | 88 (17.5%) | 141 (25.6%) | 38 (26.0%) |  |
|  | TBSA |  |  |  | <0.001 |  |  |  | <0.001 |
|  | Median [IQR] | 37 [26, 50] | 49 [32, 66] | 49 [32, 77] |  | 24 [14, 34] | 34 [20, 55] | 28 [12, 47] |  |
|  | Inhalation | 126 (43.3%) | 205 (51.2%) | 70 (50.4%) | 0.101 | 203 (40.4%) | 227 (41.3%) | 56 (38.4%) | 0.810 |
|  | LOICU |  |  |  | <0.001 |  |  |  | <0.001 |
|  | Median [IQR] | 28 [21, 40] | 28 [18, 46] | 18 [12, 34] |  | 16 [8, 26] | 27 [14, 42] | 22 [11, 40] |  |
| RDW value | Day 1 |  |  |  | <0.001 |  |  |  | <0.001 |
|  | Median [IQR] | 13.30 [12.90, 13.70] | 14.60 [14.30, 15.10] | 16.10 [15.40, 16.95] |  | 12.70 [12.30, 13.18] | 13.90 [13.50, 14.47] | 16.00 [15.43, 16.98] |  |
|  | Day 2 |  |  |  | <0.001 |  |  |  | <0.001 |
|  | Median [IQR] | 13.40 [12.90, 13.80] | 14.70 [14.40, 15.12] | 16.20 [15.60, 17.20] |  | 12.80 [12.40, 13.20] | 14.10 [13.66, 14.60] | 15.90 [15.40, 17.00] |  |
|  | Day 3 |  |  |  | <0.001 |  |  |  | <0.001 |
|  | Median [IQR] | 13.40 [13.00, 13.80] | 14.78 [14.30, 15.20] | 16.30 [15.70, 17.15] |  | 12.80 [12.40, 13.20] | 14.20 [13.76, 14.70] | 15.94 [15.43, 16.90] |  |
|  | Day 4 |  |  |  | <0.001 |  |  |  | <0.001 |
|  | Median [IQR] | 13.40 [13.00, 13.80] | 14.70 [14.38, 15.20] | 16.30 [15.80, 17.10] |  | 12.90 [12.40, 13.20] | 14.30 [13.90, 14.80] | 16.00 [15.53, 17.10] |  |
|  | Day 5 |  |  |  | <0.001 |  |  |  | <0.001 |
|  | Median [IQR] | 13.40 [13.00, 13.80] | 14.74 [14.40, 15.20] | 16.30 [15.90, 17.00] |  | 12.90 [12.40, 13.24] | 14.40 [13.90, 14.80] | 16.20 [15.50, 17.17] |  |
|  | Day 6 |  |  |  | <0.001 |  |  |  | <0.001 |
|  | Median [IQR] | 13.40 [13.00, 13.80] | 14.70 [14.39, 15.10] | 16.40 [15.90, 17.10] |  | 13.00 [12.47, 13.30] | 14.40 [14.00, 14.90] | 16.20 [15.50, 17.17] |  |
|  | Day 7 |  |  |  | <0.001 |  |  |  | <0.001 |
|  | Median [IQR] | 13.40 [13.05, 13.80] | 14.70 [14.30, 15.10] | 16.30 [15.90, 17.01] |  | 13.00 [12.50, 13.30] | 14.40 [13.99, 14.90] | 16.28 [15.52, 17.28] |  |
| Mean of overall median value | RDW |  |  |  | <0.001 |  |  |  | <0.001 |
|  | Median [IQR] | 13.40 [13.00, 13.80] | 14.70 [14.30, 15.20] | 16.30 [15.73, 17.10] |  | 12.88 [12.40, 13.20] | 14.26 [13.80, 14.70] | 16.10 [15.50, 17.10] |  |

# Fig S5. Longitudinal Bicarbonate levels Displayed in Raincloud Plots Categorized by kmlShape Package. (a. Blood Positive, b. Blood Negative)


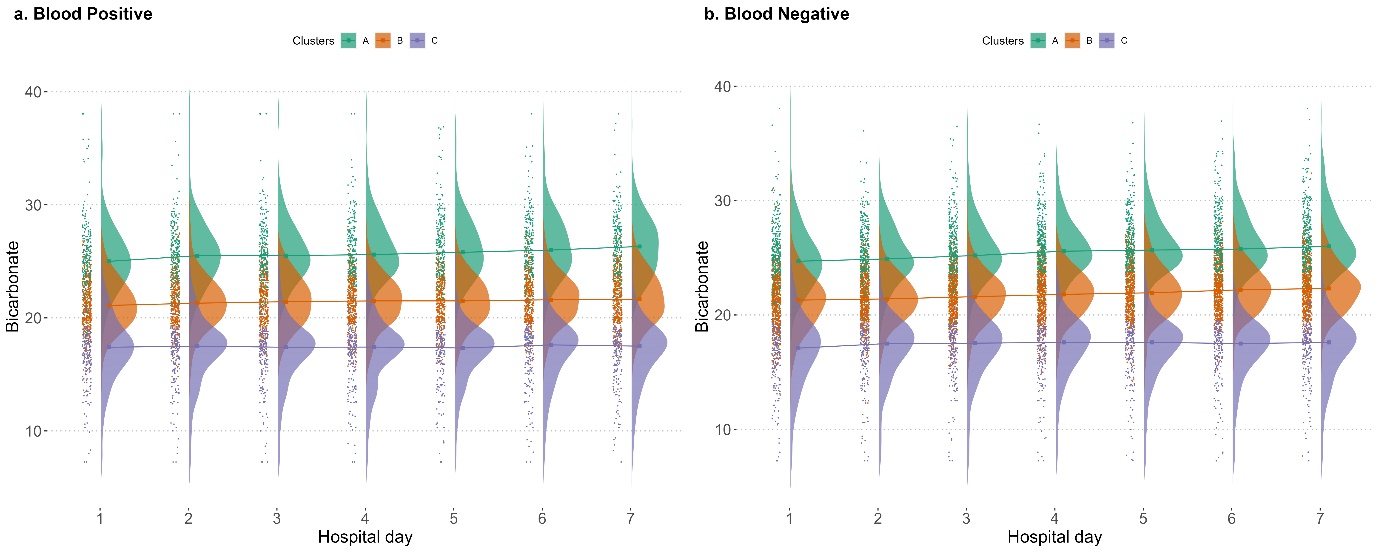


# Table S5. Characteristics and Bicarbonate levels Changes Over Time for Each Cluster in the Blood Culture-Positive/Negative Group

|  |  | Blood Positive | | | | Blood Negative | | | |
| --- | --- | --- | --- | --- | --- | --- | --- | --- | --- |
| Group | Variables | A, N = 250 (30.7%) | B, N = 365 (44.8%) | C, N = 200 (24.5%) | p-value | A, N = 380 (32.5%) | B, N = 592 (50.6%) | C, N = 199 (17.0%) | p-value |
| Demographics | Mortality | 43 (17.2%) | 144 (39.5%) | 118 (59.0%) | <0.001 | 23 (6.1%) | 128 (21.6%) | 83 (41.7%) | <0.001 |
|  | Patient Age |  |  |  | 0.065 |  |  |  | <0.001 |
|  | Median [IQR] | 52 [42, 61] | 52 [41, 62] | 55 [44, 65] |  | 50 [40, 59] | 52 [42, 64] | 56 [46, 69] |  |
|  | Sex |  |  |  | 0.790 |  |  |  | 0.725 |
|  | Male | 201 (80.4%) | 285 (78.1%) | 159 (79.5%) |  | 298 (78.4%) | 464 (78.4%) | 151 (75.9%) |  |
|  | Female | 49 (19.6%) | 80 (21.9%) | 41 (20.5%) |  | 82 (21.6%) | 128 (21.6%) | 48 (24.1%) |  |
|  | TBSA |  |  |  | <0.001 |  |  |  | <0.001 |
|  | Median [IQR] | 37 [24, 53] | 45 [32, 62] | 50 [32, 66] |  | 24 [15, 36] | 30 [18, 47] | 33 [20, 52] |  |
|  | Inhalation | 126 (50.4%) | 191 (52.3%) | 80 (40.0%) | 0.016 | 167 (43.9%) | 259 (43.8%) | 57 (28.6%) | <0.001 |
|  | LOICU |  |  |  | <0.001 |  |  |  | 0.021 |
|  | Median [IQR] | 28 [19, 41] | 29 [19, 45] | 22 [13, 37] |  | 18 [9, 33] | 22 [11, 37] | 22 [11, 36] |  |
| Bicarbonate value | Day 1 |  |  |  | <0.001 |  |  |  | <0.001 |
|  | Median [IQR] | 25.0 [23.3, 26.8] | 21.1 [19.8, 22.5] | 17.4 [15.8, 18.8] |  | 24.7 [23.2, 26.3] | 21.3 [19.7, 22.7] | 17.1 [15.2, 18.6] |  |
|  | Day 2 |  |  |  | <0.001 |  |  |  | <0.001 |
|  | Median [IQR] | 25.5 [23.8, 27.0] | 21.3 [19.9, 22.7] | 17.5 [15.9, 18.6] |  | 24.9 [23.7, 26.8] | 21.4 [20.1, 22.8] | 17.5 [15.7, 18.7] |  |
|  | Day 3 |  |  |  | <0.001 |  |  |  | <0.001 |
|  | Median [IQR] | 25.5 [24.1, 27.3] | 21.4 [19.9, 22.8] | 17.4 [15.9, 18.6] |  | 25.2 [23.9, 26.9] | 21.6 [20.3, 23.0] | 17.5 [15.9, 18.9] |  |
|  | Day 4 |  |  |  | <0.001 |  |  |  | <0.001 |
|  | Median [IQR] | 25.6 [24.2, 27.5] | 21.5 [20.0, 22.8] | 17.4 [15.9, 18.5] |  | 25.6 [24.4, 27.2] | 21.8 [20.4, 23.1] | 17.6 [15.9, 18.8] |  |
|  | Day 5 |  |  |  | <0.001 |  |  |  | <0.001 |
|  | Median [IQR] | 25.8 [24.2, 27.9] | 21.5 [20.0, 22.9] | 17.3 [15.9, 18.5] |  | 25.7 [24.5, 27.4] | 21.9 [20.6, 23.4] | 17.6 [16.1, 18.6] |  |
|  | Day 6 |  |  |  | <0.001 |  |  |  | <0.001 |
|  | Median [IQR] | 26.0 [24.4, 28.0] | 21.6 [20.1, 22.9] | 17.6 [15.4, 18.6] |  | 25.8 [24.5, 27.8] | 22.2 [20.9, 23.5] | 17.5 [15.8, 18.6] |  |
|  | Day 7 |  |  |  | <0.001 |  |  |  | <0.001 |
|  | Median [IQR] | 26.3 [24.3, 28.1] | 21.6 [20.1, 23.3] | 17.5 [15.4, 18.5] |  | 26.0 [24.8, 28.0] | 22.3 [20.9, 23.5] | 17.6 [15.7, 18.8] |  |
| Mean of overall median value | Bicarbonate |  |  |  | <0.001 |  |  |  | <0.001 |
|  | Median [IQR] | 25.6 [24.1, 27.6] | 21.4 [20.0, 22.9] | 17.5 [15.7, 18.6] |  | 25.4 [24.1, 27.2] | 21.8 [20.4, 23.2] | 17.5 [15.7, 18.7] |  |

# Fig S6. Longitudinal Haematocrit levels Displayed in Raincloud Plots Categorized by kmlShape Package. (a. Blood Positive, b. Blood Negative)


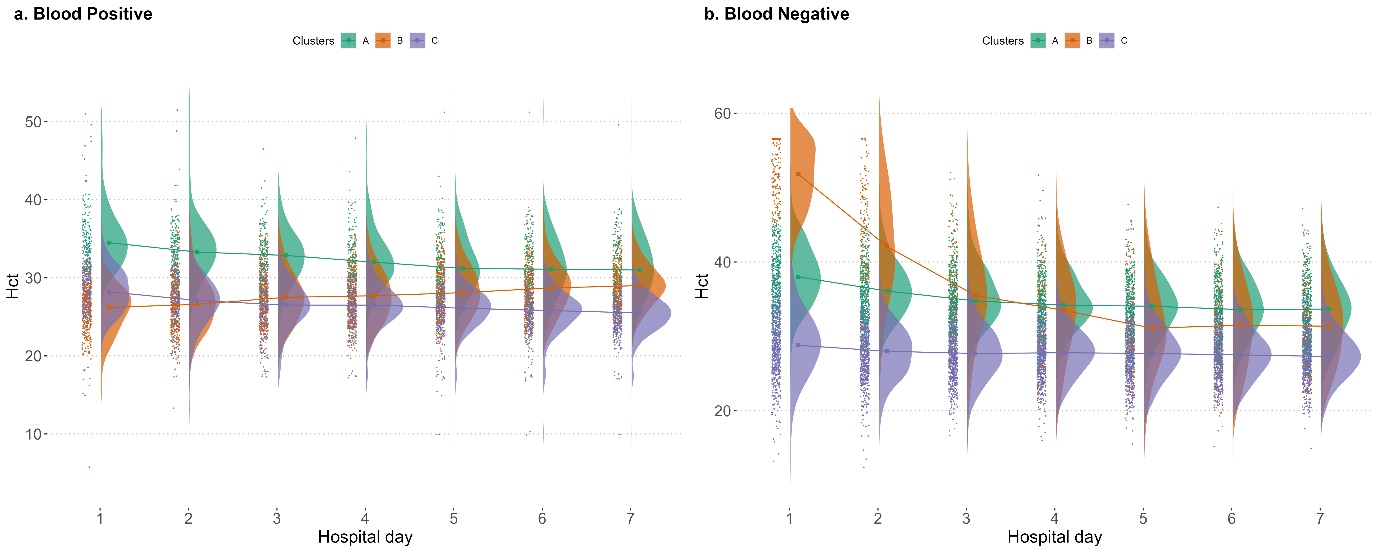


# Table S6. Characteristics and Haematocrit levels Changes Over Time for Each Cluster in the Blood Culture-Positive/Negative Group

|  |  | Blood Positive | | | | Blood Negative | | | |
| --- | --- | --- | --- | --- | --- | --- | --- | --- | --- |
| Group | Variables | A, N = 157 (18.9%) | B, N = 313 (37.7%) | C, N = 360 (43.4%) | p-value | A, N = 382 (31.9%) | B, N = 163 (13.6%) | C, N = 653 (54.5%) | p-value |
| Demographics | Mortality | 30 (19.1%) | 117 (37.4%) | 163 (45.3%) | <0.001 | 35 (9.2%) | 35 (21.5%) | 167 (25.6%) | <0.001 |
|  | Patient Age |  |  |  | 0.387 |  |  |  | <0.001 |
|  | Median [IQR] | 53 [44, 62] | 52 [43, 65] | 52 [41, 62] |  | 51 [43, 61] | 49 [42, 56] | 54 [43, 68] |  |
|  | Sex |  |  |  | 0.247 |  |  |  | <0.001 |
|  | Male | 131 (83.4%) | 240 (76.7%) | 285 (79.2%) |  | 316 (82.7%) | 142 (87.1%) | 473 (72.4%) |  |
|  | Female | 26 (16.6%) | 73 (23.3%) | 75 (20.8%) |  | 66 (17.3%) | 21 (12.9%) | 180 (27.6%) |  |
|  | TBSA |  |  |  | <0.001 |  |  |  | <0.001 |
|  | Median [IQR] | 38 [23, 52] | 42 [30, 61] | 48 [32, 65] |  | 23 [13, 38] | 35 [23, 55] | 30 [18, 46] |  |
|  | Inhalation | 74 (47.1%) | 146 (46.6%) | 181 (50.3%) | 0.614 | 156 (40.8%) | 109 (66.9%) | 221 (33.8%) | <0.001 |
|  | LOICU |  |  |  | 0.013 |  |  |  | <0.001 |
|  | Median [IQR] | 25 [16, 38] | 29 [18, 45] | 26 [16, 43] |  | 14 [7, 28] | 21 [12, 36] | 25 [13, 38] |  |
| Hct value | Day 1 |  |  |  | <0.001 |  |  |  | <0.001 |
|  | Median [IQR] | 34.5 [32.4, 36.8] | 26.2 [23.8, 27.9] | 28.2 [26.3, 30.4] |  | 38 [35, 41] | 52 [48, 56] | 29 [26, 32] |  |
|  | Day 2 |  |  |  | <0.001 |  |  |  | <0.001 |
|  | Median [IQR] | 33.3 [31.4, 35.4] | 26.6 [24.2, 28.7] | 27.1 [25.2, 29.0] |  | 36 [34, 39] | 42 [38, 48] | 28 [25, 30] |  |
|  | Day 3 |  |  |  | <0.001 |  |  |  | <0.001 |
|  | Median [IQR] | 32.9 [30.7, 34.6] | 27.5 [25.3, 29.6] | 26.5 [24.8, 28.3] |  | 34.8 [32.7, 37.8] | 35.5 [32.3, 40.3] | 27.7 [25.4, 30.1] |  |
|  | Day 4 |  |  |  | <0.001 |  |  |  | <0.001 |
|  | Median [IQR] | 32.0 [30.2, 34.1] | 27.7 [25.3, 30.0] | 26.5 [24.9, 28.0] |  | 34.2 [32.3, 37.0] | 33.5 [29.5, 36.8] | 27.8 [25.6, 30.0] |  |
|  | Day 5 |  |  |  | <0.001 |  |  |  | <0.001 |
|  | Median [IQR] | 31.2 [29.3, 33.7] | 28.1 [26.1, 30.5] | 26.1 [24.4, 27.6] |  | 34.0 [31.6, 36.8] | 31.1 [28.0, 35.3] | 27.7 [25.3, 30.0] |  |
|  | Day 6 |  |  |  | <0.001 |  |  |  | <0.001 |
|  | Median [IQR] | 31.1 [29.2, 33.8] | 28.7 [26.5, 30.6] | 25.8 [24.1, 27.2] |  | 33.6 [31.3, 36.6] | 31.5 [27.0, 34.8] | 27.5 [25.2, 29.6] |  |
|  | Day 7 |  |  |  | <0.001 |  |  |  | <0.001 |
|  | Median [IQR] | 31.0 [29.0, 34.1] | 29.0 [27.2, 30.8] | 25.5 [23.9, 26.8] |  | 33.7 [30.9, 36.4] | 31.4 [27.6, 34.6] | 27.3 [25.2, 29.4] |  |
| Mean of overall median value | Hct |  |  |  | <0.001 |  |  |  | <0.001 |
|  | Median [IQR] | 32.5 [30.2, 34.8] | 27.7 [25.4, 29.8] | 26.4 [24.7, 28.2] |  | 35.0 [32.4, 38.0] | 35.2 [30.0, 42.6] | 27.8 [25.4, 30.1] |  |

# Fig S7. Longitudinal BUN levels Displayed in Raincloud Plots Categorized by kmlShape Package. (a. Blood Positive, b. Blood Negative)


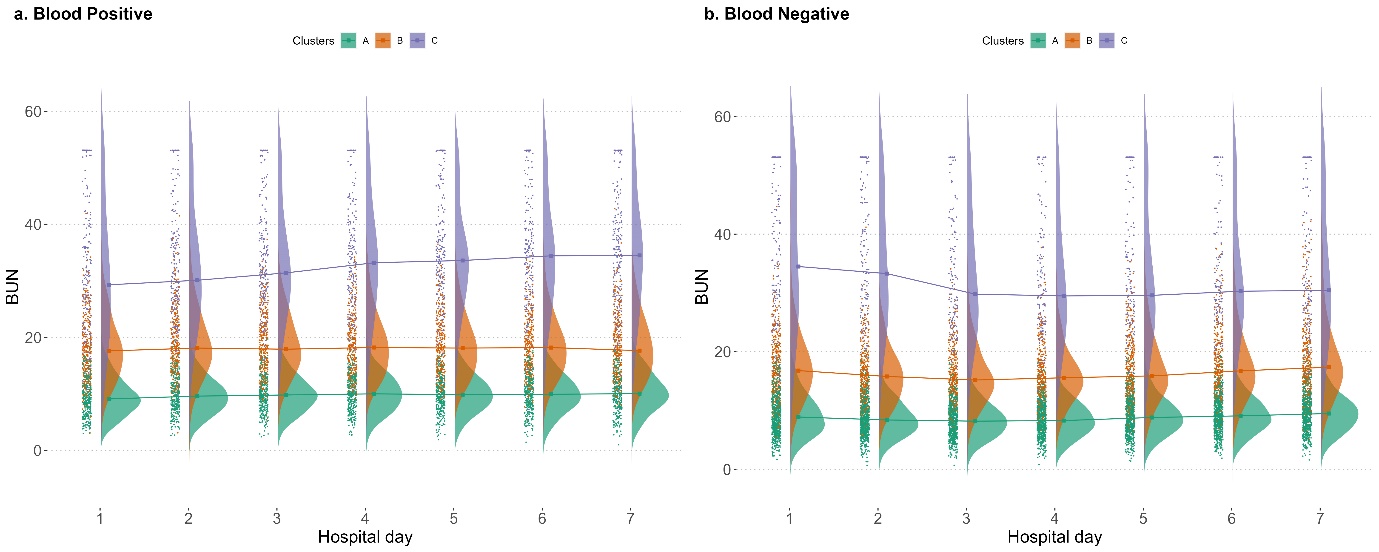


# Table S7. Characteristics and BUN levels Changes Over Time for Each Cluster in the Blood Culture-Positive/Negative Group

|  |  | Blood Positive | | | | Blood Negative | | | |
| --- | --- | --- | --- | --- | --- | --- | --- | --- | --- |
| Group | Variables | A, N = 321 (38.7%) | B, N = 304 (36.6%) | C, N = 205 (24.7%) | p-value | A, N = 701 (58.5%) | B, N = 356 (29.7%) | C, N = 141 (11.8%) | p-value |
| Demographics | Mortality | 41 (12.8%) | 110 (36.2%) | 159 (77.6%) | <0.001 | 50 (7.1%) | 102 (28.7%) | 85 (60.3%) | <0.001 |
|  | Patient Age |  |  |  | <0.001 |  |  |  | <0.001 |
|  | Median [IQR] | 48 [38, 58] | 53 [44, 64] | 56 [48, 70] |  | 48 [39, 59] | 56 [48, 67] | 61 [51, 75] |  |
|  | Sex |  |  |  | <0.001 |  |  |  | <0.001 |
|  | Male | 229 (71.3%) | 253 (83.2%) | 174 (84.9%) |  | 520 (74.2%) | 293 (82.3%) | 118 (83.7%) |  |
|  | Female | 92 (28.7%) | 51 (16.8%) | 31 (15.1%) |  | 181 (25.8%) | 63 (17.7%) | 23 (16.3%) |  |
|  | TBSA |  |  |  | <0.001 |  |  |  | <0.001 |
|  | Median [IQR] | 38 [26, 50] | 48 [31, 62] | 60 [33, 77] |  | 25 [16, 40] | 32 [20, 54] | 33 [16, 60] |  |
|  | Inhalation | 147 (45.8%) | 143 (47.0%) | 111 (54.1%) | 0.147 | 269 (38.4%) | 156 (43.8%) | 61 (43.3%) | 0.173 |
|  | LOICU |  |  |  | <0.001 |  |  |  | <0.001 |
|  | Median [IQR] | 28 [19, 40] | 33 [21, 50] | 19 [12, 33] |  | 19 [9, 33] | 26 [14, 40] | 21 [10, 34] |  |
| BUN value | Day 1 |  |  |  | <0.001 |  |  |  | <0.001 |
|  | Median [IQR] | 9 [7, 12] | 18 [14, 22] | 29 [21, 39] |  | 9 [7, 11] | 17 [14, 21] | 34 [25, 46] |  |
|  | Day 2 |  |  |  | <0.001 |  |  |  | <0.001 |
|  | Median [IQR] | 10 [7, 12] | 18 [15, 22] | 30 [25, 38] |  | 8 [6, 11] | 16 [12, 19] | 33 [26, 43] |  |
|  | Day 3 |  |  |  | <0.001 |  |  |  | <0.001 |
|  | Median [IQR] | 10 [8, 12] | 18 [15, 22] | 31 [26, 38] |  | 8 [6, 10] | 15 [13, 19] | 30 [24, 40] |  |
|  | Day 4 |  |  |  | <0.001 |  |  |  | <0.001 |
|  | Median [IQR] | 10 [8, 12] | 18 [14, 22] | 33 [26, 40] |  | 8 [7, 11] | 16 [13, 19] | 30 [24, 39] |  |
|  | Day 5 |  |  |  | <0.001 |  |  |  | <0.001 |
|  | Median [IQR] | 10 [8, 12] | 18 [14, 22] | 34 [27, 39] |  | 9 [7, 11] | 16 [13, 20] | 30 [24, 39] |  |
|  | Day 6 |  |  |  | <0.001 |  |  |  | <0.001 |
|  | Median [IQR] | 10 [8, 12] | 18 [15, 22] | 34 [27, 40] |  | 9 [7, 11] | 17 [14, 21] | 30 [26, 40] |  |
|  | Day 7 |  |  |  | <0.001 |  |  |  | <0.001 |
|  | Median [IQR] | 10 [8, 13] | 18 [14, 22] | 34 [27, 41] |  | 10 [7, 12] | 17 [14, 22] | 30 [24, 41] |  |
| Mean of overall median value | BUN |  |  |  | <0.001 |  |  |  | <0.001 |
|  | Median [IQR] | 10 [8, 12] | 18 [14, 22] | 32 [26, 39] |  | 9 [7, 11] | 16 [13, 20] | 31 [25, 41] |  |

# Fig S8. Longitudinal LD levels Displayed in Raincloud Plots Categorized by kmlShape Package. (a. Blood Positive, b. Blood Negative)


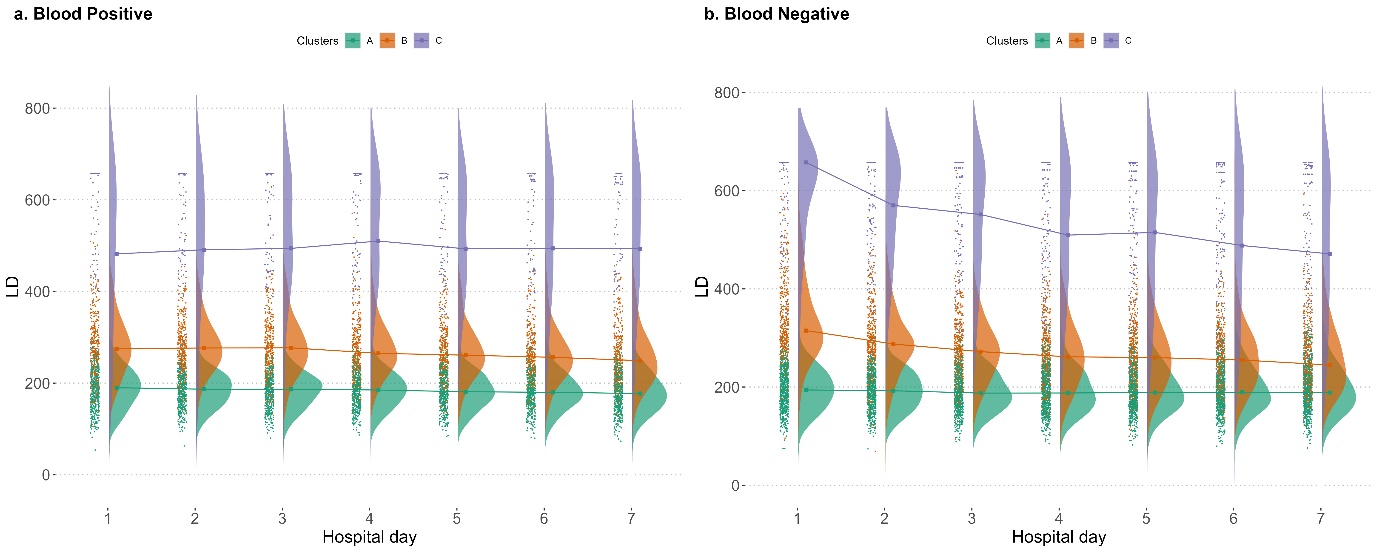


# Table S8. Characteristics and LD levels Changes Over Time for Each Cluster in the Blood Culture-Positive/Negative Group

|  |  | Blood Positive | | | | Blood Negative | | | |
| --- | --- | --- | --- | --- | --- | --- | --- | --- | --- |
| Group | Variables | A, N = 442 (53.3%) | B, N = 311 (37.5%) | C, N = 77 (9.28%) | p-value | A, N = 676 (56.4%) | B, N = 390 (32.6%) | C, N = 132 (11.0%) | p-value |
| Demographics | Mortality | 145 (32.8%) | 108 (34.7%) | 57 (74.0%) | <0.001 | 91 (13.5%) | 87 (22.3%) | 59 (44.7%) | <0.001 |
|  | Patient Age |  |  |  | <0.001 |  |  |  | 0.364 |
|  | Median [IQR] | 54 [45, 65] | 51 [39, 62] | 48 [38, 60] |  | 52 [42, 64] | 52 [43, 64] | 50 [43, 59] |  |
|  | Sex |  |  |  | 0.949 |  |  |  | 0.283 |
|  | Male | 349 (79.0%) | 247 (79.4%) | 60 (77.9%) |  | 514 (76.0%) | 313 (80.3%) | 104 (78.8%) |  |
|  | Female | 93 (21.0%) | 64 (20.6%) | 17 (22.1%) |  | 162 (24.0%) | 77 (19.7%) | 28 (21.2%) |  |
|  | TBSA |  |  |  | 0.104 |  |  |  | 0.522 |
|  | Median [IQR] | 40 [30, 60] | 44 [28, 60] | 50 [32, 76] |  | 30 [19, 42] | 26 [15, 47] | 27 [14, 48] |  |
|  | Inhalation | 214 (48.4%) | 147 (47.3%) | 40 (51.9%) | 0.761 | 263 (38.9%) | 166 (42.6%) | 57 (43.2%) | 0.422 |
|  | LOICU |  |  |  | <0.001 |  |  |  | 0.007 |
|  | Median [IQR] | 28 [18, 45] | 28 [19, 43] | 15 [10, 25] |  | 21 [11, 34] | 23 [12, 39] | 20 [6, 33] |  |
| LD value | Day 1 |  |  |  | <0.001 |  |  |  | <0.001 |
|  | Median [IQR] | 190 [157, 219] | 275 [239, 322] | 482 [352, 658] |  | 194 [165, 225] | 315 [281, 382] | 658 [509, 658] |  |
|  | Day 2 |  |  |  | <0.001 |  |  |  | <0.001 |
|  | Median [IQR] | 187 [159, 212] | 277 [243, 318] | 491 [412, 637] |  | 192 [163, 221] | 288 [253, 329] | 570 [468, 658] |  |
|  | Day 3 |  |  |  | <0.001 |  |  |  | <0.001 |
|  | Median [IQR] | 186 [158, 208] | 277 [240, 320] | 494 [438, 655] |  | 188 [161, 219] | 272 [231, 311] | 552 [432, 646] |  |
|  | Day 4 |  |  |  | <0.001 |  |  |  | <0.001 |
|  | Median [IQR] | 185 [157, 212] | 265 [237, 312] | 510 [431, 653] |  | 188 [159, 223] | 262 [218, 305] | 510 [412, 642] |  |
|  | Day 5 |  |  |  | <0.001 |  |  |  | <0.001 |
|  | Median [IQR] | 181 [157, 210] | 261 [232, 303] | 493 [423, 646] |  | 189 [161, 223] | 260 [215, 306] | 515 [380, 640] |  |
|  | Day 6 |  |  |  | <0.001 |  |  |  | <0.001 |
|  | Median [IQR] | 180 [156, 209] | 256 [224, 294] | 494 [412, 643] |  | 190 [162, 223] | 255 [214, 299] | 488 [368, 637] |  |
|  | Day 7 |  |  |  | <0.001 |  |  |  | <0.001 |
|  | Median [IQR] | 177 [150, 208] | 249 [213, 293] | 493 [412, 637] |  | 189 [159, 225] | 245 [207, 298] | 471 [344, 633] |  |
| Mean of overall median value | LD |  |  |  | <0.001 |  |  |  | <0.001 |
|  | Median [IQR] | 183 [156, 211] | 265 [231, 308] | 493 [416, 646] |  | 190 [161, 223] | 274 [227, 319] | 541 [418, 653] |  |
